# Automatic- and Transformer-Based Automatic Item Generation: A Critical Review

**DOI:** 10.3390/jintelligence13080102

**Published:** 2025-08-12

**Authors:** Markus Sommer, Martin Arendasy

**Affiliations:** Department of Psychology, University of Graz, Universitätsplatz 2, 8010 Graz, Austria

**Keywords:** test construction, automatic item generation, transformer-based automatic item generation, large language models, computerized adaptive testing, linear on-the-fly testing

## Abstract

This article provides a critical review of conceptually different approaches to automatic and transformer-based automatic item generation. Based on a discussion of the current challenges that have arisen due to changes in the use of psychometric tests in recent decades, we outline the requirements that these approaches should ideally fulfill. Subsequently, each approach is examined individually to determine the extent to which it can contribute to meeting the challenges. In doing so, we will focus on the cost savings during the actual item construction phase, the extent to which they may contribute to enhancing test validity, and potential cost savings in the item calibration phase due to either a reduction in the sample size required for item calibration or a reduction in the item loss due to insufficient psychometric characteristics. In addition, the article also aims to outline common recurring themes across these conceptually different approaches and outline areas within each approach that warrant further scientific research.

## 1. Introduction

The advent of computer-based and internet-based assessment has led to an increase in the use of summative and formative assessments. Combined with changes in best practice recommendations in educational and psychological measurement, this successively resulted in a drastic increase in item construction demands. In this article, we review and discuss several new methods of item construction that have been proposed to meet these challenges. The intention of this article is not to provide a comprehensive review, but to outline the main idea of several conceptually different methods and to discuss the extent to which each method can contribute to the construction of psychometrically sound, valid, and fair cognitive ability tests in a scalable and resource-efficient manner. To this end, we first outline the item construction demands arising from current practices in assessment to set a benchmark for evaluating and comparing various modern item construction methods. In addition, it will discuss current trends within and across different methods and outline gaps in the research literature that require further scientific research.

### 1.1. Psychometric Costs of Testing on Demand

In the last decade, it has become common to provide test-takers with multiple test-taking opportunities (cf. AERA et al. 2018; Drasgow et al. 2006; Lievens et al. 2005). However, doing so also increases the risk that test-takers attempt to memorize test items and share them with other test-takers who take the psychometric test at a later time point. These prospective test-takers may then memorize the disclosed test items to obtain higher test scores. Several studies indicate that reusing disclosed test items results in a decrease in their item difficulty and item discrimination parameters, and an increase of their local dependencies (e.g., Appelhaus et al. 2023; Joncas et al. 2018; Lievens et al. 2007; Powers 2005; Selvi 2020; Stricker 1984; Veerkamp and Glas 2000; Wagner-Menghin et al. 2013; Wood 2009; Wood et al. 2010; Yang et al. 2018; Zimmermann et al. 2016). If this item disclosure effect is ignored, the person parameter estimates will be biased. The magnitude of the bias depends on the percentage of reused disclosed test items and the magnitude of their item parameter drift (cf. Segall 2004). In addition, the psychometric tests may become more strongly correlated with measures of long-term memory (cf. Lievens et al. 2007). Thus, item disclosure compromises the construct validity of the psychometric test. Unfortunately, it also detrimentally affects the fairness of psychometric tests because test-takers have been shown to differ both in their awareness of the practice of reusing disclosed test items and in their willingness to capitalize on it by memorizing disclosed test items (cf. Powers 2005).

To facilitate testing on demand without compromising validity and fairness, test developers have turned to computerized adaptive testing (CAT: Lang 2011; Segall 2004; van der Linden and Glas 2010), multi-stage testing (MST: Zenisky et al. 2010), and multiple parallel linear on-the-fly test forms (LOFT: Folk and Smith 2002) as an alternative to classic fixed-item linear tests. Research indicates that all three test administration methods are more effective at reducing the risk of item disclosure than classic fixed-item linear tests (cf. Guo et al. 2009; Arendasy and Sommer 2017). Even though CATs have been shown to be more resistant to item disclosure than MSTs and LOFTs, the test security of the latter two methods has been shown to improve as the number of test items in each test form and the number of test forms increase.

In all three test administration methods, test security can be further enhanced by incorporating item- and content-exposure-control algorithms (cf. Georgiadou et al. 2007; Lim and Choi 2024). In addition, using multiple rotating parallel item pools further enhances test security (e.g., Ariel et al. 2006; Zhang and Chang 2005; Zhang et al. 2012). However, all three methods also drastically increase item construction demands (cf. Reckase et al. 2019). Furthermore, the initially constructed item pool needs to be constantly checked and updated (e.g., Ariel et al. 2006; Liu et al. 2019; Sinharay 2017). As a consequence, test developers need to be able to construct a substantial number of psychometrically sound test items with predictable item parameters and content specifications during the entire lifespan of the psychometric test.

### 1.2. Psychometric Costs of Retesting

Similar to testing on demand, retesting is often conceived as an important aspect of the fair and valid use of psychometric tests by test-takers (cf. AERA et al. 2018; Lievens et al. 2005). Unfortunately, several meta-analyses (cf. Calamia et al. 2012; Hausknecht et al. 2007; Kulik et al. 1984a; Scharfen et al. 2018) indicate that retesting leads to an increase in test-takers’ scores. The effect size of retest-score gains depends on various factors such as the cognitive ability domain measured, test-takers’ general mental ability, the time period between the initial test and retest, the number of retests taken, and the specific kind of retest form used. In general, retest score gains are more pronounced (mean effect sizes: 0.37 to 0.42) when using identical retest forms compared to alternate retest forms (mean effect sizes: 0.22 to 0.23). However, if test items used in the alternate retest form only differ in surface features from those used at the initial test administration, retest score gains are often almost identical to those in identical retest forms (cf. Arendasy and Sommer 2013a, 2017; Matton et al. 2011; Morley et al. 2004). Furthermore, retest effects are larger if the time interval between initial test administration and retest is short. However, they remain notable even with time intervals of several years (cf. Burke 1997). In addition, retest effects tend to increase with the number of retests taken until a plateau is reached. The number of retest sessions needed to reach that plateau differs across cognitive ability domains measured (cf. Calamia et al. 2012; Hausknecht et al. 2007; Kulik et al. 1984a; Scharfen et al. 2018). Furthermore, retest score gains are more pronounced for high-g test-takers than for low-g test-takers (cf. Kulik et al. 1984a).

Several theoretical models have been proposed to explain the observed score gains due to retesting (for a detailed discussion, see Arendasy and Sommer 2017; Lievens et al. 2007). One of the most prominent explanations is the *strategy refinement hypothesis*. The strategy refinement hypothesis posits that test-takers use various solution strategies that differ in their effectiveness and efficiency (e.g., Bethell-Fox et al. 1984; Fehringer 2020; Heil and Jansen-Osmann 2008; Heil et al. 1998; Jarosz and Wiley 2012; Liu et al. 2023b, 2025; Verguts and De Boeck 2002; Vigneau et al. 2006). The solution strategies are stored in procedural long-term memory together with information on their effectiveness and efficiency given certain task characteristics (e.g., Anderson et al. 1997; Siegler 1996). When solving test items with a lower person-specific difficulty (e.g., person-specific *p* ≥ .70), test-takers have sufficient cognitive resources left to update information on the effectiveness and efficiency of a solution strategy given certain task characteristics. This allows them to gradually tailor their solution strategy choices to the task demands, which leads to an increase in narrower test-specific cognitive abilities. This hypothesis is consistent with studies indicating that test-takers are able to learn to tailor their solution strategies to task demands during test-taking even with little to no feedback (e.g., Fehringer 2023; Hayes et al. 2015; Heil et al. 1998; Ren et al. 2012; Verguts and De Boeck 2002).

The strategy refinement hypothesis explains several key findings regarding the size of the retest effect. First, since the items of the initial test and an identical retest have more item design features in common, this fosters transfer compared to alternate retest forms, which share fewer item design features with the initial test items (e.g., Anderson et al. 1997; Siegler 1996). Second, the assumption that strategy refinement is a gradual process explains why the number of retests administered has been shown to affect the size of the retest effect. The same explanation also accounts for the finding that high-g test-takers benefit more from retesting than low-g test-takers. High-g test-takers encounter more items with a person-specific lower difficulty while working on a fixed-item linear test than do low-g test-takers. They therefore have more opportunities to learn to refine their solution strategies (Arendasy and Sommer 2017). Third, the hypothesis that strategy refinement leads to a gradual increase in a narrower test-specific cognitive ability is consistent with research findings indicating that retesting neither induces differential item functioning at the item level nor alters the structural relations between the cognitive ability tests and their relation to psychometric g or other higher-order traits (e.g., Arendasy and Sommer 2013a, 2017; Estrada et al. 2015; Reeve and Lam 2005). This is due to strategy refinements leading to a gradual improvement in narrower cognitive abilities. Last but not least, the model also posits that these improvements in narrower cognitive abilities should not generalize to higher-order traits. This final prediction, deduced from the strategy refinement hypothesis, is consistent with research findings indicating that retest score gains do not seem to generalize to higher-order traits (e.g., Arendasy and Sommer 2013a, 2017; Estrada et al. 2015; Krautter et al. 2021; Levacher et al. 2021; Loesche et al. 2015; Matton et al. 2011; Reeve and Lam 2005; Schneider and Sparfeldt 2021a, 2021b; Schneider et al. 2020; te Nijenhuis et al. 2007).

The strategy refinement hypothesis also has implications for how to reduce retest effects. Usually, CATs administer items of higher (*p* = .50) person-specific difficulty to all test-takers (cf. Arendasy and Sommer 2017; Guo et al. 2009). This should not only prevent strategy refinement during test-taking but also equalize the learning opportunities between high-g and low-g test-takers. As a consequence, high-g test-takers, who take a CAT at the initial test administration session, should no longer benefit more from retesting than their low-g peers. This prediction is consistent with recent research findings indicating that CAT indeed reduces retest effects when administered at the initial test administration session (Arendasy and Sommer 2017). However, the authors also showed that retest effects still exist when test-takers take a fixed-item linear retest form at the initial test administration session, followed by either a CAT or an alternate fixed-item linear test form. This indicates that test-takers may learn to refine their solution strategies in-between test-taking sessions by practicing sample test items, which are commonly used in test preparation.

### 1.3. Psychometric Costs of Test Preparation

Due to the increased use of psychometric tests, the test preparation industry has become a lucrative business on its own (cf. Buchmann et al. 2010; Powers 2012; Zwick 2002). Prospective test-takers are often offered a variety of different test preparation methods from commercial and/or non-commercial providers. Researchers (e.g., Briggs 2009; Messick 1982; Powers 2012) distinguish between three methods of test preparation: test familiarization, practice-based training, and test coaching.

*Test familiarization* merely aims to familiarize test-takers with the mechanics of test-taking to reduce construct-irrelevant variance due to test familiarity and test anxiety. This method of test preparation does not intend to improve test-takers’ test scores. Consistent with this aim, research shows that test familiarization increases test-wiseness and reduces test anxiety, but its effect on test-takers’ test scores is negligible (cf. Burns et al. 2008; Powers and Alderman 1983). For this reason, cost-free test familiarization has become a standard practice and is often seen as mandatory by the test-takers (cf. AERA et al. 2018; Denker et al. 2023; Lievens et al. 2005).

*Practice-based training*, on the other hand, constitutes an informal test-taker-driven test preparation method that aims at improving test-takers’ test scores. During practice-based training, test-takers practice sample-test items with or without feedback to improve their test scores (cf. Briggs 2009; Messick 1982; Powers 2012). Several meta-analyses (cf. Bangert-Drowns et al. 1983; Hausknecht et al. 2007; Kulik et al. 1984a; Messick and Jungeblut 1981) and independent studies (e.g., Burns et al. 2008; Hermes et al. 2019; Hermes et al. 2023; Weppert et al. 2023) indicate that practice-based training improves test-takers’ test scores (Cohen’s d = 0.22 to 0.40). The size of the practice-based training effect varies with the similarity of the sample-test items and the actual test items (cf. Arendasy and Sommer 2013a, 2017; Freund and Holling 2011; Morley et al. 2004), the general mental ability of the test-taker (cf. Kulik et al. 1984a), the amount of time and effort invested into practicing (cf. Bangert-Drowns et al. 1983; Becker 1990; Hermes et al. 2019; Kulik et al. 1984b; Messick and Jungeblut 1981), and the specific cognitive ability domain trained (e.g., Arendasy et al. 2016; Hermes et al. 2019, 2023; Kulik et al. 1984a; Weppert et al. 2023).

*Test coaching*, on the other hand, is a formal instructor-driven test preparation method that consists of the following components aimed to improve test-takers’ test scores: test familiarization, practice-based training, formal instruction and feedback, and instruction on specific test-taking strategies (cf. Allalouf and Ben-Shakhar 1998; Becker 1990; Briggs 2009; Messick 1982; Powers 2012). Similar to practice-based training, several meta-analyses and large-scale studies (e.g., Becker 1990; Briggs 2009; Bangert-Drowns et al. 1983; Kulik et al. 1984b; Lilly and Montgomery 2011; Messick and Jungeblut 1981; Powers and Rock 1999; Witt 1993; Weppert et al. 2023) indicate that test coaching increases test-takers’ test scores (Cohen’s d = 0.33 to 0.64). The size of the score gains depends on the general mental ability of the test-taker (cf. Kulik et al. 1984b), the time devoted to practicing (cf. Appelrouth et al. 2017; Becker 1990; Messick and Jungeblut 1981), the specific cognitive ability trained (e.g., Becker 1990; Briggs 2009; Bangert-Drowns et al. 1983; Kulik et al. 1984b; Powers and Rock 1999), and the feedback provided (cf. Krautter et al. 2021). The added value of test coaching on its own, compared to practice-based training, is small and has been attributed to the effect of feedback and formal instruction (cf. Appelrouth et al. 2017; Krautter et al. 2021; Levacher et al. 2021; Loesche et al. 2015; Schneider and Sparfeldt 2021a, 2021b) in addition to a more optimal pacing of test preparation (cf. Appelrouth et al. 2017). However, combining test coaching with practice-based training has been shown to lead to a notable increase in test scores.

Thus, both practice-based training and test coaching are effective at improving test scores. Measurement invariance analyses across test-takers differing in the amount and kind of test preparation (e.g., Arendasy et al. 2016; Estrada et al. 2015; Hermes et al. 2019; Levacher et al. 2021; Krautter et al. 2021; Sommer et al. 2025) indicate that individual differences in test preparation neither induce uniform nor non-uniform differential item functioning (DIF) at the item level, nor do they alter the structural relationship between the subtests and the higher-order traits that they have been constructed to measure. Nevertheless, this line of research also clearly shows that score gains do not generalize to higher-order traits. They are confined to the narrower test-specific cognitive abilities trained (cf. Arendasy et al. 2016; Hermes et al. 2019; Sommer et al. 2025). This implies that—similar to retest effects—test preparation-based score gains may reflect an increase in narrower test-specific abilities due to strategy refinement. This conclusion is corroborated by an eye-movement study, which showed that test-takers indeed resorted to more effective and efficient solution strategies after receiving a tutorial on how to solve the test items (Loesche et al. 2015, experiment 4).

Although test preparation does not detrimentally affect measurement invariance and construct validity, it potentially threatens fairness if test-takers differ in their access to test preparation (cf. Borsboom et al. 2008; Buchmann et al. 2010; Sommer et al. 2025). While studies using previous birth cohorts of test-takers (e.g., Buchmann et al. 2010; Park and Becks 2015) indicated that test-takers whose parents are more educated and wealthier had more access to test preparation than their peers, studies conducted with more recent birth cohorts (e.g., Arendasy et al. 2016; Lee et al. 2023a; Sommer et al. 2025) failed to replicate this finding. A possible explanation for this finding is that universities and commercial companies started to provide test-takers with either less costly or even cost-free test preparation opportunities to ensure equal opportunities to perform for all test-takers (cf. AERA et al. 2018; Arendasy et al. 2016; Campion et al. 2019; Hermes et al. 2019; Lee et al. 2023a; Sommer et al. 2025). However, this practice also extends the item construction demands beyond the constructing and maintaining of item pools for assessment.

### 1.4. Implications for Test Construction

The research outlined above has implications for the construction of test items and sample-test items. Test developers need to be able to construct large item pools that are optimal for their intended use in terms of the shape and distribution of the item parameters, and in terms of the content specifications. Several methods have been proposed to construct blueprints for optimal item pools (e.g., He and Reckase 2014; Reckase 2010; Veldkamp and van der Linden 2010). The drawback of these methods is that they assume that test developers can construct test items with the pre-specified item parameters and content specifications. In the case of cognitive ability tests, content specifications refer to item design features and combinations thereof. Item design features hypothesized to affect the item parameters are called *radicals* (Irvine 2002), while non-salient item design features (=surface features) that do not affect the item parameters are referred to as *incidentals* (Irvine 2002). The item pools should not only contain information on the item parameters themselves but also information on the combination of radicals and incidentals used to construct each test item to facilitate the use of item- and content-exposure control algorithms (cf. Georgiadou et al. 2007; Gierl et al. 2022a; Lim and Choi 2024).

To minimize item disclosure, test developers should be able to construct test items whose item parameters not only span the entire range of the person parameter distribution but also mimic its shape and distribution (cf. Reckase 2010; Veldkamp and van der Linden 2010). Furthermore, to construct multiple rotating parallel item pools (e.g., Ariel et al. 2006; Zhang et al. 2012), test developers need to be able to construct multiple instances of all test items with identical radical combinations (and item parameters) but different incidentals (Irvine 2002). These kinds of test items are referred to as *item isomorphs* (Bejar 2002) or item clones (Glas and van der Linden 2003). Furthermore, to support item- and content-exposure control (e.g., for an overview, see Georgiadou et al. 2007; Gierl et al. 2022a; Lim and Choi 2024), test developers should also be able to construct test items that exhibit statistically identical item parameters, despite using a different combination of radicals, at all segments of the latent trait continuum. These kinds of test items have been referred to as *psychometrically matched instances* (cf. Arendasy and Sommer 2013a) and are a prerequisite for content exposure control to work as intended.

Item construction demands further increase if the test developers aim to reduce individual differences in access to test preparation by providing cost-free test preparation (e.g., AERA et al. 2018; Arendasy et al. 2016; Campion et al. 2019; Hermes et al. 2019; Lee et al. 2023a; Sommer et al. 2025). According to the standards for educational and psychological assessment (cf. AERA et al. 2018), sample-test items used in test preparation should resemble the actual test items in terms of the item format and item construction principles. This implies that they should be representative of the actual test items in terms of the distribution of their item parameters and also mirror them in terms of their affordances and constraints (Greeno et al. 1993) for construct-related solution strategies. As a result, sample-test items need to be representative for—but not identical to—the actual test items in terms of their radical combinations (Irvine 2002). Furthermore, if test preparation includes feedback and tutorials, these tutorials should also be representative and cover all difficulty levels (cf. Arendasy et al. 2016; Krautter et al. 2021; Sommer et al. 2025). This requires test developers to be able to construct not only multiple item pools intended to be used for assessment, but also one or more item pools consisting of sample-test items that can be used in cost-free practice-based training. Furthermore, since test preparation increases test scores without generalizing to higher-order traits (cf. Section 1.3), test developers need to be able to shift the spectrum of the item difficulties upward to counteract retest and test preparation effects.

The specifications of ideal item pools for assessment and training purposes outlined above are thus rather specific and go beyond merely being able to generate large pools of test items. They can therefore also be used as a benchmark to evaluate and compare conceptually different methods of item construction in terms of their potential to meet these requirements in a cost-efficient and scalable manner.

## 2. Human-Constructed Test Items (H-IG)

Traditional item construction involves trained human item writers, who are also subject-matter experts in the domain covered by the psychometric test (cf. AERA et al. 2018; Hornke and Habon 1986; Lane et al. 2016). The test development process usually starts with a definition of the latent trait to be measured and the selection of an item format that can be used to measure the latent trait. Next, subject-matter experts outline the cognitive processes hypothesized to be involved in solving the test items, in addition to other content-matter specifications. This is done to provide a more detailed specification of what needs to be achieved during item writing. Once this is done, human item writers construct test items on an item-by-item basis using either their experience and expertise or a blueprint of the item construction rationale (cf. AERA et al. 2018; Hornke and Habon 1986; Lane et al. 2016). Next, the constructed test items are submitted to a panel of independent subject-matter experts for review. This review process covers both subject-matter considerations (e.g., factuality check, etc.) and a fairness review (for details, see AERA et al. 2018; Lane et al. 2016). Once the test items have passed through this review process, they are edited and revised if necessary. In the latter case, the items are again submitted to review. This cycle continues until the test items are deemed to be of sufficient quality by the panel of subject-matter experts. Next, the test items are passed on to field testing to estimate their item parameters, and to evaluate their dimensionality and fairness using descriptive item response theory modeling (cf. De Boeck and Wilson 2004). This brief description of the human item writing process nicely illustrates that item writing is a complex process that requires a considerable amount of financial, time, and human resources. Furthermore, since test items are constructed on an item-to-item basis, this process is hardly scalable. It is therefore not surprising that item construction has been referred to as a critical bottleneck in test construction (cf. Drasgow et al. 2006; Kosh et al. 2019; Wainer 2002; Zickar 2020).

In addition, there is evidence that writing test items requires a lot of expertise and requires continuous training. For instance, Webb et al. (2015) showed that subject-matter experts trained in item writing constructed fewer test items, which were identified as flawed in a subsequent review process, compared to subject-matter experts without any training in item writing. However, a short one-time training in item construction does not seem to suffice (Gupta et al. 2020). Repeated and continuous training is required to construct test items that pass the review process and also exhibit acceptable classical item difficulty and classical item discrimination parameters in subsequent field tests (e.g., Beg et al. 2021; Lee et al. 2024). This result is consistent with research findings indicating that expertise is important in human item writing (e.g., Eleragi et al. 2025; Jozefowicz et al. 2002; Nemec and Welch 2016) and that building expertise through repeated and continuous training and feedback improves the quality of human item writing (cf. Karthikeyan et al. 2019). By contrast, lack of motivation and time pressure reduce the quality of the human-made test items (cf. Karthikeyan et al. 2019). Although—to the best of our knowledge—we currently lack studies examining item writer-related determinants of the quality of human-made items for intelligence tests, it seems plausible that the above-cited results obtained in formative and summative assessment in medical education generalize to the construction of intelligence tests.

Furthermore, there is evidence that human item writers also have difficulties predicting the empirically estimated difficulty of the test items they constructed. Several studies (e.g., Attali et al. 2014; Bejar 1983; Berenbon and McHugh 2023; El Masri et al. 2017; Holmes et al. 2018; Impara and Plake 1998; Rogausch et al. 2010; Sayın and Bulut 2024; Sayın and Gören 2023; Sydorenko 2011; Wonde et al. 2024) indicate that the correlation between empirically estimated item difficulties and item difficulties predicted by subject-matter experts ranges from ≤0.10 to 0.80. One interesting finding from these studies is that the precision of subject-matter experts’ judgement improves with feedback. Subject-matter experts become more accurate at predicting the empirically estimates difficulty the more they learn about the results of actual empirical studies and theoretical models that link item design features to cognitive processing demands. Thus, using theory and research helps human item writers to tailor their item writing more to the demands of the specifications of the blueprint for an ideal item pool and to avoid the use of non-functional distractors that often lead to a misfit of the chosen descriptive IRT model in field testing. However, providing item writers with this information requires conducting research on predictors of the difficulty of test items in the first place. Nonetheless, even with sufficient knowledge in these areas, human item writers still experience difficulties constructing and accurately predicting the difficulty of more difficult test items that exceed their own standing on the latent trait (cf. Hornke and Habon 1986; Sayın and Gören 2023). This line of research nicely illustrates that solely relying on the expertise of human item writers is no viable short-cut to theory-building and empirical studies. Providing detailed specifications of the item construction rationale, as well as theory and research findings on the cognitive processes involved in item solving, helps human item writers to improve the quality of the constructed test items.

Although it is possible to construct psychometrically sound test items using human item writers by combining theory, research, and subject-matter expertise, the item construction process cannot be scaled. This is due to the fact that test items are still constructed and reviewed on an item-to-item basis. This particular feature of human item writing (H-IG) is at odds with the demands for a large number of test items within a short amount of time (cf. Drasgow et al. 2006; Kosh et al. 2019; Wainer 2002; Zickar 2020). The costs associated with the continuous item construction process are often referred to as an explanation for why the anticipated cost saving from moving from fixed-item linear paper-pencil tests to CATs have not been realized thus far (cf. Luecht 2005).

Therefore, researchers and test developers have turned to alternative methods of item construction, which have been shown to be more scalable due to a higher degree of automation during the item construction process (cf. Falcão et al. 2023, 2024; Kosh et al. 2019). The approaches used to achieve this aim are referred to as rule-based automatic item generation (AIG: Arendasy and Sommer 2011; Embretson and Yang 2007; Irvine and Kyllonen 2002) and transformer-based automatic item generation (TB-AIG: Attali et al. 2022; von Davier 2019), respectively. Both methods can be summarized under the umbrella term automatic item generation. The distinction between AIG and TB-AIG resembles the theoretical distinction between the use of symbolic models, on the one hand, and connectionist models, on the other hand, in cognitive modeling research (cf. Klahr and MacWhinney 1997; van der Maas et al. 2021). It also has important consequences on how each method can be used in practice. For ease of reference, we will use the shortcut automatic item generation (AIG) to refer specifically to rule-based automatic item generation in all following sections.

## 3. Automatic Item Generation (AIG)

Automatic item generation (AIG: Arendasy and Sommer 2011, 2012a; Arendasy et al. 2024; Embretson and Yang 2007; Irvine and Kyllonen 2002) is a collective term for various methods using pre-programmed algorithms to automatically construct large numbers of test items with predictable item parameters. This is done by systematically varying a set of radicals (Irvine 2002) and incidentals (Irvine 2002). The historical roots of AIG can be traced back to the 1970s in the United States (cf. Bejar 2002; Drasgow et al. 2006) and Europe (cf. Arendasy and Sommer 2011). In this article, we use the term automatic item generation in a more narrow, non-technological sense to exclusively refer to rule-based automatic item generation, which uses computer programs programmed from scratch to construct test items using symbolic models. AIG is often incorrectly portrayed as a unified approach (cf. Gierl et al. 2022a; von Davier 2018, 2019). In fact, there are three conceptually different approaches to automatic item generation. The difference concerns (1) the extent to which the selection of radicals is based on a theoretical model (weak- vs. strong theory), (2) the extent to which radicals are varied independently (schema-based vs. element-based), (3) the extent to which the item generation process is automated (human-made vs. semi-automated vs. automated), and (4) the inclusion of a quality control component (single- vs. dual-component item generation), which checks the factual correctness of the scoring key and also aims to reduce the likelihood of differential item functioning (DIF) and construct-irrelevant variance. Differences in these four characteristics between various approaches to AIG not only affect the validity evidence that can be advanced to support test score interpretations, but also the cost savings at various points in the item construction process (cf. Arendasy and Sommer 2011; Kosh et al. 2019).

### 3.1. Item Model Approach

In the *item model* approach (e.g., Bejar 2002; Choi and Zhang 2019; LaDuca et al. 1986), the construction of the item generator starts with the selection of one or more templates. These templates (cf. Mislevy and Riconscente 2006) are also referred to as item models (Bejar 2002), schemata (Arendasy and Sommer 2007), item shells (Glas and van der Linden 2003), or parent items (Roid and Haladyna 1982). For ease of communication, we will use the term item model since it has been used more commonly than other terms. Item models constitute the basic building blocks of the item generator and are usually hard coded into the item generator. Each item model consists of fixed and variable elements. The variable elements of an item model can either consist of a list of alternatives for incidentals or a list of alternatives for radicals (Gierl et al. 2012; Glas and van der Linden 2003). All items generated on the basis of a single item model are called instances (Graf et al. 2005) or siblings (Sinharay et al. 2003). Items constructed by only manipulating incidentals are referred to as *item isomorphs* (Bejar 2002) or item clones (Glas and van der Linden 2003). Item isomorphs are hypothesized to exhibit invariant (statistically identical) item parameters. By contrast, items constructed by manipulating radicals are called *item variants* (Bejar 2002). Item variants are hypothesized to differ in their item parameters and therefore exhibit more within-item model variation in their item parameters than item isomorphs (cf. Bejar 2002; Geerlings et al. 2013; Glas et al. 2016; Glas and van der Linden 2003; Sinharay et al. 2003; Sinharay and Johnson 2008). In the item model approach, radicals are not varied independently of each other. In fact, each item model is characterized by a specific combination of radicals and radical levels, which are hard coded in the item model.

Item models can be constructed in three different ways, which differ in their theoretical foundation. The first two means rely solely on the subject-matter expertise and intuition of the item-model writers and are therefore referred to as the weak theory approach (Drasgow et al. 2006). This is because even experienced subject-matter experts may not always correctly guess what makes a particular test item more or less difficult and what kind of misconceptions test-takers may hold that lead them to select specific distractors (cf. Attali et al. 2014; Bejar 1983; Berenbon and McHugh 2023; Holmes et al. 2018; Impara and Plake 1998; El Masri et al. 2017; Rogausch et al. 2010; Sayın and Bulut 2024; Sayın and Gören 2023; Sydorenko 2011; Wonde et al. 2024).

Within the weak theory approach, item models can be constructed on the basis of one or more operational test items, which are inspected by subject-matter experts to identify commonalities and differences in order to determine item design features that can be systematically combined and manipulated (cf. Bejar 2002; Fu et al. 2022). Alternatively, item models can be constructed from scratch by asking item model writers to define elements that can be systematically varied within each item model and to propose permissible values for these elements based on their subject-matter expertise (cf. Gierl and Lai 2012).

The third alternative is to construct item models based on pre-existing cognitive processing models for a specific item format (cf. Enright et al. 2002; Graf et al. 2005). The main idea here is to replace the subject-matter expertise and intuition of the item-model writers with an empirically validated cognitive processing model that specifies item design features hypothesized to be linked to cognitive processing demands. The main benefit of this method is that it builds on empirically validated theoretical models, which increase the construct validity of the resulting test items provided that these cognitive processes and the item design features linked to them have been shown to explain the item parameters of the test items (cf. Arendasy and Sommer 2011, 2012a; Arendasy et al. 2024; Embretson and Yang 2007). For this reason, the third approach is often referred to as the strong theory approach (Drasgow et al. 2006).

The item model approach focuses heavily on the item model itself as its central building block. Psychometric modeling often boils down to examining the extent to which item isomorphs exhibit invariant item parameters (e.g., Fu et al. 2022) and examining the extent to which variability between item variants can be adequately modeled by means of expected item family functions (cf. Geerlings et al. 2013; Glas et al. 2016; Sinharay et al. 2003; Sinharay and Johnson 2008; Zorowitz et al. 2024). Validating the radicals by means of explanatory item response theory modeling is often not considered mandatory in the item model approach. While proponents of the strong theory approach acknowledge the benefits of explanatory item response theory modeling, proponents of the weak theory approach (e.g., Gierl et al. 2022b) often argue that calibrating the item models and examining their content validity by means of subject-matter expert ratings are sufficient to provide the necessary validity evidence in high-stakes settings.

The main reason for this focus is that the item model approach aims to reduce item construction costs not only by scaling the item construction process itself but also by shifting the focus of item calibration from the individual test items themselves to the item models. That is, instead of calibrating hundreds of test items, test developers merely need to calibrate the item models and model the variation of the item parameters within each item model by means of expected item family functions. Since each item model reflects a unique combination of radical levels, the number of item models is much lower than the number of item instances that can be generated by an item model. If calibrating item models instead of individual test items works, this can result in notable cost savings. Several simulation studies (e.g., Bejar et al. 2002; Colvin et al. 2016; Glas and van der Linden 2003; Someshwar 2024; Tian and Choi 2023) indicated that although using calibrated item families and expected item family functions instead of the actual item parameters of the item instances introduces a small bias in the person parameter estimate, this effect can be offset by administering a few additional test items. Thus, the approach of calibrating item models instead of individual test items seems feasible and can result in notable cost reduction, provided that the within-family variation is low and can be adequately modeled. Unfortunately, this is not always the case (e.g., Fu et al. 2022; Zorowitz et al. 2024).

The item model approach currently is the most popular approach. This has led some researchers (e.g., Circi et al. 2023; Gierl et al. 2022a; von Davier 2018) to equate the item model approach with the entire field of automatic item generation. Embretson and Yang (2007) explained the popularity of the item model approach with two of its features. On the one hand, the item model approach can be used to construct item models with a stronger or weaker theoretical foundation. On the other hand, item models are convenient to implement in programming terms. Thus far, the item model approach has been successfully used by the Educational Testing Service (ETS) in admission testing (cf. Bejar 2002), the construction of test items for the medical licensing exam in Canada (e.g., Gierl et al. 2012), high-stakes pharmacy exams (e.g., Leslie and Gierl 2023), test items measuring misconceptions in physics (e.g., Wancham et al. 2023), Turkish literature knowledge test items (e.g., Sayin and Gierl 2023), and to construct an open-source cognitive test (Ryoo et al. 2022), among various other examples.

Across different practical applications, the number of item models exhibiting a unique combination of radical levels varied from k = 6 (Ryoo et al. 2022) to k ≈ 60 (e.g., Gierl et al. 2012). Given that the number of item models constitutes an upper limit for the number of different item parameters, the number of psychometrically distinct items is limited by the number of item models implemented into the item generator. This affects the feasibility of constructing multiple item pools that can be used for assessment and practice-based training. In particular, constructing psychometrically matched test items may become more difficult with a lower number of item models. This is unfortunate because a combination of isomorphic and psychometrically matched test items is needed to simultaneously construct item pools for assessment and practice-based training to enhance fairness without compromising validity (cf. Section 1.4).

Some authors (e.g., Gierl et al. 2022a; Glas and van der Linden 2003; Yi et al. 2008) also discuss additional measurement and test security challenges associated with the use of item models. For instance, using item models in CATs requires content coding and content exposure control to ensure that isomorphic test items are not administered within the same test administration session. Doing so would lead to a local stochastic dependency of the item responses and a corresponding bias in the person-parameter estimate (cf. Glas and van der Linden 2003; Segall 2004). Furthermore, if an item model has been disclosed or leaked, all items generated by that item-model are compromised. This can lead to biased person-parameter estimates (cf. Yi et al. 2008). Thus, appropriate technical test- or item model security measures have to be taken to protect the item models from disclosure and organized item theft (for an overview, see Foster 2016).

A final concern relates to the number of test items that have to be discarded after the item calibration phase due to insufficient psychometric characteristics (e.g., misfit of the 2PL model or the 1PL Rasch model to the data). In most applications that examined the dimensionality of the automatically generated test items, the percentage of item loss ranged from 10 to 30 percent. In addition, item models constructed using weak theory often require substantial modifications after the expert reviews and calibration phase to reduce the likelihood that item instances include non-functional distractors or other item-writing flaws (e.g., Falcão et al. 2023, 2024; Gierl and Lai 2012; Lai et al. 2016). Similar to item loss, the number of required revision rounds must be taken into account when evaluating potential cost savings (cf. Kosh et al. 2019).

### 3.2. Cognitive Design System Approach

The cognitive design system approach (cf. Embretson 1998, 2002, 2016; Embretson and Yang 2007; Gorin 2006) extends the item model approaches based on strong theory. It heavily resorts to cognitive science research to provide a more detailed and precise definition of the latent cognitive ability trait in terms of the cognitive processes hypothesized to be involved in solving the test items. In this sense the, cognitive design system approach is similar to the evidence-centered design system approach proposed by Mislevy and associates (cf. Mislevy et al. 2003). Embretson and associates (cf. Embretson 2002, 2005; Embretson and Yang 2007; Gorin 2006) recommend starting the construction of an item generator with such a precise definition, which is referred to as a *cognitive item model*. Ideally, this cognitive item model has been implemented as a computational model. Computational models are symbolic and/or connectionist computer programs that not only specify the cognitive processes hypothesized to be involved in solving certain test items but also outline the link between these cognitive processing demands and the item design features that affect them. These item design features serve as radicals (Irvine 2002) in item generation. The behavior of computational models can be compared to human problem solvers as a feasibility check on whether the theoretical model may constitute a possible data-generating mechanism (cf. Farrell and Lewandowsky 2010; Fried 2020; Guest and Martin 2021; Klahr and MacWhinney 1997; Smaldino 2020; van der Maas et al. 2021). The main benefit of implementing verbal theories into a computational model is that this process forces the scientists to reason more precisely about the latent traits that they intend to measure (cf. Farrell and Lewandowsky 2010; Fried 2020; Guest and Martin 2021; Smaldino 2020). Thus, test developers using computational models ultimately operate with a more precise definition of the latent trait to be measured.

The drawback of using computational models to deduce radicals is that such models may not exist in all cognitive ability domains. In such cases, Embretson and associates suggest examining test-takers’ solution strategies using eye-movement analyses, verbal protocols, etc., to deduce hypotheses on cognitive processes involved in problem solving and how these cognitive processes are linked to item design features (for an overview, see Embretson 2016). Next, these hypotheses should be tested by means of explanatory item response theory modeling of existing psychometric test data (e.g., Baldonado et al. 2015; Daniel and Embretson 2010; Draheim et al. 2018; Embretson 1998, 2002, 2023; Embretson and Daniel 2008; Embretson and Gorin 2001; Embretson and Kingston 2018; Gorin 2005; Gorin and Embretson 2006; Ivie and Embretson 2010; Kaller et al. 2011; O’Reilly et al. 2018; Svetina et al. 2011). Evaluating the status of radicals and incidentals by means of explanatory item response theory modeling is also highly recommended in cases in which computational cognitive item models are available. The main argument is that the radicals and corresponding cognitive processes should be able to account for a large percentage of the variance in the item parameters if the cognitive item model constitutes a possible data-generating mechanism (cf. Embretson 1998, 2016; Embretson and Yang 2007). The intention behind combining computational cognitive modeling with explanatory item response theory modeling is to *maximize the construct-related variance in the item parameters* by systematically manipulating item design features linked to cognitive processing demands, which are central to the definition of the latent cognitive ability trait. However, Embretson and associates note that radicals (Irvine 2002) are often confounded in existing psychometric tests. This complicates the interpretation of the research findings due to multi-collinearity (cf. Fischer 1995). Therefore, the next step consists of the construction of an automatic item generator, in which radicals—and combinations thereof—are more systematically varied, and the use of explanatory item response theory modeling (cf. De Boeck and Wilson 2004) to examine the extent to which the item parameters of the automatically generated test items can be predicted by the radicals. In most studies using the cognitive design system approach, the correlation between the empirically estimated item difficulty parameters and those predicted on the basis of the radicals (Irvine 2002) varied from R = 0.70 to R = 0.90. Thus, the radicals accounted for 49 to 80 percent of the variance in the item difficulty parameters of the respective descriptive item response theory model (e.g., 1PL Rasch model or 2PL model).

In a nutshell, the cognitive design system approach shifts the expenses in terms of financial, time, and human resources to the initial test construction phases. This strategy pays off if it increases the psychometric quality of the automatically generated items in terms of their fit to the chosen descriptive item response theory model (e.g., 1PL Rasch model, 2PL model, etc.) and a lack of differential item functioning (DIF), and in terms of the variance in the item parameters explained by the radicals (cf. Kosh et al. 2019). Several simulation studies (e.g., Doebler 2012; Embretson 1999; Freund et al. 2008; Mislevy et al. 1993; Matteucci et al. 2012; Someshwar 2024) indicated that if the variance in the item parameters accounted for by the radicals is sufficiently high (R^2^ ≥ 0.80), the effect of using predicted item parameters instead of empirically calibrated item parameters on the precision and bias of the person parameter estimate becomes negligible and can be easily offset by the administration of a few additional test items. Interestingly, Someshwar (2024) recently showed that using item parameters predicted by an explanatory item response theory model such as the Linear Logistic Test Model (LLTM; Fischer 1995) is superior to using expected item family functions in terms of the precision and bias in the person parameter estimates. This indicates that the strong focus on theory in the cognitive design system approach not only has the potential to provide additional validity evidence (cf. AERA et al. 2018) but it can also surpass the potential cost savings of the item model approach. In addition, using explanatory item response theory modeling has also been shown to help explain the intended or unintended variance of the item parameters within item models (cf. Cho et al. 2014; Geerlings et al. 2013; Glas et al. 2016; Sinharay et al. 2003; Sinharay and Johnson 2008). This valuable information can be used to either refine the item models or adjust the item parameters of the automatically generated items accordingly.

Thus far, the cognitive design system approach has been successfully used to automatically generate figural matrices (e.g., Embretson 1998, 2002; Freund et al. 2008; Primi 2002, 2014), analogies (e.g., Blum and Holling 2018; Bejar et al. 2012), number series (e.g., Sun et al. 2019), propositional reasoning items (e.g., Gühne et al. 2020), spatial ability test items (e.g., Fehringer 2020; Ivie and Embretson 2010; Shi et al. 2023), mathematical ability test items (e.g., Daniel and Embretson 2010; Embretson and Kingston 2018; Enright et al. 2002; Holling et al. 2009), perceptual speed test items (e.g., Doebler and Holling 2016), and to semi-automatically generate reading comprehension test items (e.g., Förster and Kuhn 2023; Gorin 2005). Most researchers chose to implement radical combinations by constructing item models due to the ease of programming implementation. The number of item models ranged from 30 to 60 across studies. Since researchers chose to use item models, considerations regarding test security for the cognitive design system approach are almost identical to the ones outlined for the item model approach (cf. Section 3.1, seventh paragraph). Thus, the extent to which the cognitive design system approach can deal with the increased test item demands depends on the number of item models, their distribution along the latent ability continuum, and whether it is possible to generate psychometrically matched item models differing in their radical combinations along the entire latent trait continuum. Like the item model approach, researchers using the cognitive design system approach often reported an item loss of 10 to 30 percent after the item calibration phase due to either a misfit to the 2PL model or differential item functioning. This item loss also has to be taken into account when evaluating cost savings (cf. Kosh et al. 2019).

### 3.3. Automatic Min-Max Approach

To address the issue of item loss after the item calibration phase, Arendasy and associates (cf. Arendasy 2004; Arendasy and Sommer 2011, 2012a; Arendasy et al. 2024) developed the automatic min-max approach. The automatic min-max approach can be seen as a further development of the cognitive design system approach. Like Embretson and associates (cf. Embretson 1998, 2016; Embretson and Yang 2007; Gorin 2006), the automatic min-max approach combines cognitive science research, individual differences research, and explanatory item response theory modeling in constructing and evaluating an item generator. Like the cognitive design system approach, it is heavily rooted in modern validity theory and cognitive science (cf. Arendasy and Sommer 2011, 2012a; Arendasy et al. 2024).

The automatic min-max approach resorts to individual differences research to provide an initial definition of the latent cognitive ability trait and its location within current models of human intelligence. Next, the cognitive processes involved in solving the test items are specified in more detail by resorting to cognitive processing models that ideally have been implemented as computational models. Like the cognitive design system approach, the automatic min-max approach also emphasises the theoretical and practical value of computational models in constructing a *cognitve model* that serves as the basis for the selection of the radicals (Irvine 2002). However, it deviates from the cognitive design system approach by proposing that researchers should not solely resort to a single computational model. Instead, Arendasy and associates (cf. Arendasy 2004; Arendasy and Sommer 2011, 2012a; Arendasy et al. 2024) recommended using and comparing multiple computational models. This is because most existing computational models have been primarily tested on a particular item set or a particular item format (for a similar argument, see Yang and Kunda 2023; Yang et al. 2022). Thus, using a single computational model makes it more difficult to separate cognitive processes and corresponding item design features that are central to the core of the definition of the latent trait from those that are more specific to either a certain item format or a certain set of test items (cf. Arendasy and Sommer 2011, 2012a; Arendasy et al. 2024). Ideally, hypotheses deduced in this manner should be empirically tested by means of psychometric experiments. To illustrate this point, we will give two examples. Arendasy and Sommer (2012b) examined item design features that have been hypothesized to moderate the size of gender differences in figural matrices despite not being central to the definition of inductive reasoning. Using several established and experimentally designed figural matrices tests in addition to number series, the authors demonstrated that some non-central item design features used in figural matrices increase the likelihood of gender-related differential item functioning. Furthermore, performance differences in psychometric tests using these item design features did not generalize to a higher-order trait reflecting individual differences in inductive reasoning. Based on these results, the authors concluded that these item design features are not central to the definition of inductive reasoning. Similarly, Arendasy and associates (e.g., Arendasy 2000; Arendasy and Sommer 2010; Arendasy et al. 2010; Arendasy et al. 2010) tested the hypothesis that gender differences in complex three-dimensional mental rotation are in part due to differences in the ability to utilize depth cues using psychometric experiments involving 3D shutter glasses. Unlike the figural matrices reasoning example, differences in the use of depth cues are central to the definition of complex three-dimensional mental rotation according to several computational models. However, one may need to constrain the values of this radical to avoid differential item functioning across gender without compromising construct representation (cf. Arendasy and Sommer 2010). These two examples nicely illustrate that the selection of radicals (Irvine 2002) and the choice of an item format should be well founded empirically and theoretically.

Once the radicals have been selected, the cognitive model is applied to the chosen item format. The manipulation of the radicals in this *cognitive item model* focuses on the systematic manipulation of radicals (Irvine 2002) that affect cognitive processes central to the definition of the latent trait. By contrast, item design features linked to non-central cognitive processing demands are constrained at a certain value to ensure that they neither induce differential item functioning nor affect the item parameters. This is done to *maximize the construct-related variance* in the item parameters.

If no prior computational cognitive (item) model is available, the automatic min-max approach proposes using studies on test-takers’ solution strategies in combination with explanatory item response theory modeling to deduce hypotheses on potential radicals (Irvine 2002) and to gradually formulate a cognitive (item) model (cf. Arendasy and Sommer 2011, 2012a; Arendasy et al. 2024). This recommendation is identical to the cognitive design system approach. We briefly outline how this can be done using two examples. Sommer et al. (2009) combined cognitive task analysis and explanatory item response theory modeling to deduce a set of radicals (Irvine 2002) from the test items of an existing tachistoscopic traffic perception test to enable the construction of a new item pool. The hypotheses deduced in this earlier stage were subsequently tested using data obtained with the new semi-automatically constructed test items that were analyzed with the Linear Logistic Test Model (LLTM; Fischer 1995). This approach was chosen because, at that time, no computational models of perceptual speed were available for tachistoscopic tests. Similarly, Arendasy et al. (2024) resorted to computational models for the Tower of London (ToL) and supplemented prior research on cognitive processes involved in ToL problem solving with studies using retrospective verbal reports obtained during scheduling problem solving to deduce a set of radicals (Irvine 2002) that can be used to automatically generate items measuring planning ability using scheduling problems as an item format. Thus, the utilization of the automatic min-max approach is not limited to a strong theory approach. However, the authors emphasize that the strength of validity arguments that can be formed differs between a strong and a weak theory approach.

Similar to the cognitive design system approach (cf. Embretson 1998, 2002, 2016; Embretson and Yang 2007; Gorin 2006), validating the radicals by means of explanatory item response theory models (for an overview, see De Boeck and Wilson 2004) is mandatory in the automatic min-max approach. This is because the radicals constitute the basic building blocks of the item generator in the automatic min-max approach (element-based approach). Thus, instead of implementing item models that exhibit a fixed combination of radicals and radical levels, Arendasy and associates suggested implementing radicals and algorithms on how to combine them. This allows test developers to manipulate the radicals (Irvine 2002) independently of each other. Although the element-based approach is more difficult to implement from a programming perspective, it enhances test security by complicating item theft (cf. Arendasy and Sommer 2011). Despite this advantage, the authors also note that, in some cases, a schema-based approach may be more appropriate. This is the case in algebra word problem solving (cf. Arendasy and Sommer 2007; Arendasy et al. 2006). The main reason for a schema-based approach in the case of algebra word problems is that it mimics the cognitive representation of algebra word problems in the minds of the test-takers.

Up to this point, the automatic min-max approach only differs gradually from the cognitive design system approach. The most important difference between these two approaches resides in the use of a separate quality control component. Arendasy and associates (cf. Arendasy 2004; Arendasy and Sommer 2011, 2012a; Arendasy et al. 2024) argued that researchers should explicitly outline cognitive processes involved in solving the test items that are hypothesized to be construct-unrelated but may be triggered by certain item design features. These item design features are referred to as functional constraints (Greeno et al. 1993). The quality control component aims to minimize construct-unrelated variance in the item parameters and to reduce the likelihood of differential item functioning by suppressing the use of functional constraints during item generation. In order to prevent the risk of construct-underrepresentation, functional constraints need to be empirically validated (for examples, see Arendasy 2004; Arendasy and Sommer 2005, 2010, 2012b, 2013b; Arendasy et al. 2012). Arendasy and associates showed that using a quality control component minimizes item loss due to insufficient psychometric characteristics. Across several cognitive ability domains, the authors report an item loss of ≤1 percent despite the use of more restrictive IRT models such as the 1PL Rasch model (for a replication, see Hines 2017). This not only translated into cost savings (cf. Kosh et al. 2019) but also effectively leads to a more precise definition of the latent trait (cf. Farrell and Lewandowsky 2010; Fried 2020; Guest and Martin 2021; Smaldino 2020).

Once the radicals (Irvine 2002) and functional constraints (Greeno et al. 1993) have been implemented into the item generator, large numbers of test items can be automatically generated. Item generators constructed using the automatic min-max approach have been used to generate number series (e.g., Arendasy and Sommer 2012a), figural matrices and analogies (e.g., Arendasy and Sommer 2005, 2012b; Hines 2017), syllogisms (e.g., Arendasy et al. 2020), algebra word problems (e.g., Arendasy and Sommer 2007; Arendasy et al. 2006), arithmetic fluency items (e.g., Arendasy et al. 2007), computational estimation items (e.g., Arendasy et al. 2020), complex three-dimensional mental rotation items (e.g., Arendasy and Sommer 2010; Arendasy et al. 2010; Arendasy et al. 2011), visualization items (e.g., Arendasy et al. 2020), multi-lingual verbal fluency items (e.g., Arendasy et al. 2012), synonym items (e.g., Arendasy et al. 2020), and planning ability items (e.g., Arendasy et al. 2024). In addition, the automatic min-max approach has also been used to semi-automatically construct reading comprehension items (e.g., Arendasy et al. 2020) and perceptual speed items (e.g., Sommer et al. 2009). In all cases, the Linear Logistic Test Model (LLTM; Fischer 1995) has been shown to fit the data obtained with a roughly representative set of k = 120 to k = 320 test items exhibiting unique radical combinations reasonably well. The correlation between the empirically estimated item parameters and the ones predicted on the basis of the LLTM basic parameter estimates of the radicals (Irvine 2002) varied from R = 0.89 to R = 0.96 (for an overview, see Arendasy et al. 2020; Hines 2017). Thus, cognitive processes linked to the radicals account for 72 to 92 percent of the variance in the item- and person parameter estimates. This finding not only provides empirical evidence on the construct validity of the automatically generated test items but it also indicates that cost saving may be possible.

Several simulation studies (e.g., Doebler 2012; Embretson 1999; Freund et al. 2008; Mislevy et al. 1993; Matteucci et al. 2012; Someshwar 2024) indicated that the effect of using predicted item parameters instead of calibrated item parameters on the precision and bias of the person parameter estimate becomes negligible and can be easily offset by the administration of a few additional test items if the predicted and calibrated item parameters are highly correlated (R ≥ 0.90). Based on these results, using predicted item parameters instead of calibrated item parameters would be possible. Doing so would lead to a drastic reduction in the item calibration costs to develop and update multiple item pools that can be used in CATs and practice-based training. The number of items that can be generated with the item generators also seems to be sufficiently large. Furthermore, there is evidence that the item generators are able to construct psychometrically matched test items along large parts of the latent trait continuum (cf. Arendasy and Sommer 2013a). The high correlation between empirically estimated item parameters and those predicted by the LLTM-basic parameter estimates also enables the automatic generation of test items on-the-fly during the administration of a CAT. This has been referred to as either adaptogenic testing (Arendasy 2004) or on-the-fly item generation (Bejar et al. 2002). To our knowledge, this option has only been realized once as a beta version (Arendasy 2004). In addition to technical considerations (e.g., time needed for automatic item generation, implementation of an item generator, etc.), we currently still lack empirical data and theoretical models that would allow us to estimate whether such an approach would be accepted by test-takers and decision-makers (cf. Arendasy 2004).

Based on the research findings outlined above, the automatic min-max approach seems to largely meet the requirements to keep up with the increased item construction demands outlined in Section 1.4. However, the extent to which this is the case varies across item generators. In addition, as discussed above, not all item generators are able to generate test items fully automatically. For instance, the item generator used to construct reading comprehension test items is only semi-automatic (cf. Arendasy et al. 2020). Human intervention, and in some cases a collaboration between human item writers and transformer networks (cf. Section 4.1), was needed to iteratively construct the reading passages from scratch based on content- and linguistic specifications provided by the item generator. Due to its semi-automatic nature, the costs are naturally higher than for item generators in the other cognitive ability domains that are fully automated.

## 4. Transformer-Based Automatic Item Generation (TB-AIG)

More recently, several researchers (e.g., Attali et al. 2022; Bulut et al. 2024; Hao et al. 2024; Lee et al. 2023b; von Davier 2018, 2019) proposed including large language models in the toolbox of test developers as a subvariant of AIG. The rapid progress in natural language processing due to the introduction of transformer networks (cf. Vaswani et al. 2017) has enabled large language models to handle a variety of natural language processing tasks (e.g., translation, text summarization, question answering, and writing text) at a level similar to humans. This sparked the interest of professionals working in computer science and psychometrics, who started examining the feasibility of transformer networks in item generation (for reviews: Kurdi et al. 2020; Song et al. 2025; Tan et al. 2024). Reviews on TB-AIG noted that most studies examined the feasibility of using transformer networks in the verbal ability domain as well as the domains of medicine and computer science. Furthermore, most studies merely examined whether TB-AIG items are rated similarly to items constructed by human item writers during the item review process. Few studies included results on the calibration of the TB-AIG items. Thus, at present, there is a lack of data that allows us to examine the extent to which TB-AIG can contribute to meeting the increased item construction demands. What we know thus far is that TB-AIG is feasible and that it reduces the time needed to construct the test items.

However, cost savings during the item construction process are smaller compared to AIG. Advocates of TB-AIG (e.g., Attali et al. 2022; Bulut et al. 2024; Hao et al. 2024; von Davier 2019) recommend keeping human subject-matter experts in the loop during the entire item construction process. This implies that item generation has to be semi-automatic. The reason for this recommendation has to do with the perils and pitfalls arising from the architecture of transformer networks and how they are trained prior to their use. In the next section, we will provide a brief description of transformer networks. Next, we will discuss concerns regarding the use of transformer networks in item construction without human oversight (for an overview, see Hao et al. 2024) before discussing how transformer networks have been used in psychological and educational assessment.

### 4.1. Introduction to Transformer Networks

Transformer networks are the successors of recurrent neural networks (for a brief overview, see Lee et al. 2023b; von Davier 2019). Their main benefit is that they can process information in parallel and can more accurately capture distant semantic relations between words and text. The architecture of transformer networks consists of a combination of encoder- and decoder blocks (cf. Vaswani et al. 2017). The encoder extracts features from the input, while the decoder uses information received from the encoder in conjunction with its learning database to produce an output.

Both encoder and decoder consist of multiple stacks of units organized into layers. Each encoder unit in the first layer receives the input, which has been converted into tokens (numerical representations of words, etc.) and enriched with position coding. The encoder unit converts the input into a vector, which reflects the meaning of the token in a multidimensional space. These vectors are commonly referred to as word embeddings. To ensure that long-distance relations between tokens are adequately captured, the transformer network uses a self-attention mechanism, which pays more attention to tokens that are jointly more relevant to the meaning of the input. The word embedding vectors go through position-wise feed-forward layers for further transformation by being handed over to the units in subsequent layers. This part of the transformer architecture is similar to neural networks. Although the linear transformations from one layer to another are identical, each layer of the feed-forward network uses different parameters that have been optimized in the pre-training phase (cf. Brown et al. 2020; Vaswani et al. 2017; Yang et al. 2024). In a final step, the optimized embedding is handed over to the decoder.

Similar to the encoder, the decoder also consists of multiple layers of decoder units and self-attention mechanisms. The encoder and decoder are connected to each other via a cross-attention mechanism. Based on the input of the encoder, the decoder predicts the next token as an output on the basis of the probability of that token given the input from the encoder and the tokens in the text corpus it has been trained on. The predicted token is included into the sequence of tokens to create an updated vector, which conditions the next token to be generated by the decoder until the task is finished.

Based on this brief description of transformer networks, transformer networks can be seen as probabilistic models that produce output based on the conditional probability of a token given the input, the training data, and all previously generated tokens that surround it (cf. Sobieszek and Price 2022).

The various transformer networks differ in their focus on the encoder side, the decoder side, or the encoder and decoder side. Decoder models and encoder–decoder models have dominated in recent years (Yang et al. 2024). These two types of transformer networks differ regarding how their model parameters have been optimized. Encoder-decoder models use an unsupervised learning paradigm, in which randomly selected parts of the training data are masked, and the transformer network has to predict the mask token. By contrast, the parameters of decoder transformer networks are optimized by means of an unsupervised learning paradigm, in which they have to predict the next token in the training data given all previously encountered tokens (cf. Yang et al. 2024).

Both unsupervised learning paradigms enable the transformer network to learn the conditional probabilities of tokens from large training data sets, which often consist of billions of text data points. However, the specific learning paradigm affects the kind of transfer tasks on which the models can be expected to excel (e.g., Yang et al. 2024). Prominent examples of decoder models include the Generative Pre-trained Transformer 3 (GPT-3: Brown et al. 2020) and its successor GPT-4 (OpenAI 2023) and LLaMA (Touvron et al. 2023). Examples of encoder–decoder models include the Bidirectional Encoder Representations from Transformers (BERT: Devlin et al. 2018) and the Text-to-text Transfer Transformer (T5: Raffel et al. 2020). The four examples above have been chosen because they were frequently used in item generation studies (cf. Kurdi et al. 2020; Song et al. 2025; Tan et al. 2024). Currently available transformer networks also differ regarding the number of parameters that have been optimized, the tokenizers used, and the scope, quality, diversity, distribution, and specific content and language of the training and validation data (Yang et al. 2024). All of these characteristics are known to affect the performance on subsequent transfer tasks (cf. Yang et al. 2024; Zha et al. 2025). Thus, researchers should choose a transformer network with those characteristics in mind. In practical applications, this often comes down to the need to use different transformer networks for specific sub-tasks during item generation.

Once the model parameters have been optimized, the transformer network can be applied to various transfer tasks by either fine-tuning its parameters through training on that specific transfer task or by in-context learning via prompts.

In the former case, the pre-trained transformer network receives additional training on a specific transfer task (e.g., solving math word problems, text classification, etc.) with feedback (supervised learning). This leads to an update of the parameters of the transformer model. The benefit of this approach is that it improves performance on the transfer task. However, fine-tuning requires large annotated high-quality training examples and sufficient computing power—especially when fine-tuning larger transformer models—to update its parameters (Brown et al. 2020).

In-context learning via prompts, on the other hand, does not alter the parameters of the pre-trained transformer network. Instead, the transformer network is presented with a precise task description, possibly in addition to a few selected examples, and explanations (e.g., Ahn and Yin 2025; Al Faraby et al. 2024; Bozkurt and Sharma 2023; Heston and Khun 2023; Lee et al. 2023c; Li and Zhang 2024; Liu et al. 2023a; Reynolds and McDonell 2021; Sahoo et al. 2024; Schulhoff et al. 2024). This approach is referred to as k-shot learning or few-shot learning, where k is the number of examples provided to the transformer network. The main finding from studies examining prompting techniques is that the quality, structure, and clarity of the prompts, as well as the quality, distribution, and representativeness of the examples, affect the quality of the output. Furthermore, even slight paraphrases of a prompt may sometimes yield qualitatively different output.

This effect is due to how transformer networks work. They generate tokens based on the conditional probability given the tokens in the input (here, the prompts of the user) and the tokens in their training data. Thus, different prompts naturally lead to different conditional probabilities for the output. This also explains the finding that an identical prompt may yield different output depending on the specific transformer network used (for an illustrative example, see Säuberli and Clematide 2024). This is because transformer networks may not only differ in the number of parameters and how they have been optimized; they may also differ in the scope, quality, diversity, distribution, and specific content of their training data.

Furthermore, the research cited above indicates that prompts often need to be revised and optimized iteratively. This process is referred to as either prompt engineering or iterative prompting. Prompt engineering can be facilitated by implementing a collaborative platform that enables human item writers to iteratively test, revise, and refine their prompts based on the output generated by the transformer network to ensure that its final output meets their expectations (cf. Reza et al. 2024).

In a nutshell, transformer networks learn subject-matter domain knowledge and linguistic knowledge by inferring regularities and probabilities between tokens (e.g., words, numbers, etc.) in their training data in terms of conditional probabilities given all tokens that surround them (Sobieszek and Price 2022).

### 4.2. Perils of Using Transformer-Based Automatic Item Generation in a Fully Automated Manner

Proponents of TB-AIG propose a semi-automatic item generation approach that keeps human subject-matter experts in the loop during the entire test item construction process (e.g., Attali et al. 2022; Bulut et al. 2024; Hao et al. 2024; von Davier 2018, 2019). The main reasons for this proposal are linked to how transformer networks work.

The first concern regarding the use of TB-AIG without human oversight concerns the *factual correctness of the output* (cf. Bulut et al. 2024; Hao et al. 2024). In constructing cognitive ability test items, it is important that the response(s) scored as the correct answer(s) are indeed factually correct. Research indicates that transformer networks sometimes have difficulties identifying the factually correct answer(s). Two recent meta-analyses (Liu et al. 2024; Waldock et al. 2024) examined whether transformer networks such as GPT and BERT are able to solve United States Medical Licensing Exam (USMLE) items. Liu et al. (2024) summarized k = 45 studies using GPT to solve USMLE items conducted between January 2022 and May 2024. The authors report an overall accuracy rate of 81% [CI: 78%; 84%] for GPT-4 and 58% [CI: 53%; 63%] for GPT-3.5. Waldock et al. (2024) conducted a meta-analysis of k = 32 studies published until September 2023. They report an overall accuracy rate of 51% [CI: 46%; 56%] for GPT-2 and GPT-3 and 64% [CI: 60%; 67%] for ChatCPT (GPT-3.5). Based on these results, newer versions of GPT outperform their precursors. This finding is attributable both to an increase in the number of model parameters and the size of the training data and to the use of previous prompt data in training the successor transformer networks. Furthermore, these meta-analyses indicate that the accuracy of picking the correct answer decreases as the test items become more difficult. However, fine-tuned prompting generally improves accuracy across all difficulty levels. In addition, transformer models have been shown to be inconsistent in their selections of the correct answer alternative when prompted to do so multiple times using the same prompt. This problem was more apparent in older versions than in newer versions of the same transformer network. However, even the more recent version of the transformer networks chose the correct answer consistently in ≤80% of the cases. The above-cited meta-analytic findings were replicated in subsequent studies on USMLE items and in studies using items from other medical or nursing licensing exams (e.g., Bhayana et al. 2023; Funk et al. 2024; Riedel et al. 2023; Su et al. 2024). Similar results were obtained for measures of verbal and numerical reasoning commonly used in educational admission testing in the United States (cf. Abu-Haifa et al. 2024; Hickman et al. 2024), math problems (for an overview, see Lu et al. 2022), and natural science and STEM problems (e.g., Chan et al. 2025; Schulze Balhorn et al. 2024). These results are not surprising because transformer networks have been trained to produce probable output given the constraints of the prompts and the training data. Despite being probable, plausible, and in some cases even convincing, the output does not necessarily have to be factually correct (cf. Sobieszek and Price 2022). This implies that human subject-matter experts need to examine the output and fact-check the content of the test items. This conclusion is consistent with recent research evidence indicating that TB-AIG-generated items often exhibit more than one possible correct answer despite using elaborated state-of-the-art prompts. By contrast, items constructed by means of AIG do not exhibit this problem (e.g., Chung and Kim 2024; Emekli and Karahan 2025). However, the two studies cited above also indicate that human item writers sometimes unintentionally construct items with more than one correct answer if the item exceeds their own standing on the latent trait. This may indicate that, in addition to content reviews by human subject-matter experts, a more rule-based approach may be required to ensure that all constructed test items align with their scoring key.

*Copyright and ethical issues* are an additional concern (cf. Bulut et al. 2024; Hao et al. 2024). In Section 4.1 (third paragraph), we outlined that the parameters of transformer networks have to be optimized in a pre-training phase. This is done by means of different unsupervised learning paradigms (e.g., masked token prediction or next token prediction) using billions of text data scraped from the internet, various corpora, and private sources. The precise content of these data is often not outlined in detail. This makes it difficult to determine whether the training data contained copyrighted data and the extent to which the output is based on it. Various studies (e.g., Lee et al. 2022; McCoy et al. 2023) indicated that the output of transformer models may indeed contain verbatim and/or paraphrased plagiarism of their training data. Although this does not happen often, it cannot be ruled out. Thus, test developers need to check whether the content of the TB-AIG test items violates copyright claims.

The third concern is also linked to the training data of the transformer networks. Data used to optimize the parameters of the transformer network may include *stereotypes about different groups* of people. This may lead to the presence of a socio-demographic bias in the output of the transformer network. Various studies (e.g., Balestri 2025; Kamruzzaman et al. 2024; Luca et al. 2025; Ranjan et al. 2024; Thakur 2023) indicated that both closed (commercial) and open-source transformer networks have been trained with data that contain stereotypes and biases against selected groups. Thus, fairness and bias reviews by human subject-matter experts are needed to ensure that the final test items are not biased. However, such fairness reviews may require a sufficiently large and diverse team of subject-matter experts. Belzak et al. (2023) recently showed that both TB-AIG test items and human-written items may contain a bias leading to DIF despite extensive fairness reviews by subject-matter experts. One means to deal with this issue is to use explanatory item response theory modeling (for an overview, see De Boeck and Wilson 2004) to examine whether the source of DIF can be traced down to certain radicals or other item design features (e.g., Arendasy and Sommer 2013b; Rajeb et al. 2024; Yu 1994) in addition to fairness reviews. Results from such studies can be used both in subsequent item generation and in the continuous training of subject-matter experts.

Taken together, these findings indicate that TB-AIG items need to be reviewed and refined by subject-matter experts prior to item calibration. Thus, the advice to keep human subject-matter experts in the loop during the entire item construction process is warranted (cf. Bulut et al. 2024; Hao et al. 2024; Sobieszek and Price 2022). As a consequence, TB-AIG is only feasible in a semi-automatic manner.

### 4.3. Studies Using Transformer-Based Automatic Item Generation in the Cognitive Ability Domain

Despite these limitations, TB-AIG has the potential to considerably reduce the amount of time and effort in item construction. TB-AIG seems particularly useful in cognitive ability domains, where the aforementioned AIG approaches have so far been difficult to implement in a fully automatic manner. Examples include measures of vocabulary and reading comprehension as well as knowledge tests. Several recent reviews (cf. Kurdi et al. 2020; Song et al. 2025; Tan et al. 2024) indicated that most studies using TB-AIG were indeed conducted in these cognitive ability domains. Furthermore, the reviews indicated that in most articles the TB-AIG items were solely validated by means of subject-matter expert ratings. Examinations of the psychometric characteristics of TB-AIG test items have been rare. If psychometric characteristics were reported at all, researchers often utilized classical test theory instead of item response theory modeling. Thus, at present, there is limited data on the extent to which TB-AIG can contribute to meeting the increased item demands outlined in Section 1.4. In addition, some reviews (cf. Song et al. 2025; Tan et al. 2024) also noted a trend toward the use of a weak theory approach. We agree with all these points. In addition, we noticed another important trend. While some authors incorporate ideas of the *item model approach* (Bejar et al. 2002) into their prompts, others use ideas inspired by an *element-based approach* (Arendasy and Sommer 2011). In the next sections, we will outline research conducted within both approaches to TB-AIG in the domains of verbal ability and medical clinical reasoning.

#### 4.3.1. Item Model-Based TB-AIG

Proponents of an *item model-based approach to TB-AIG* argue that item models (Bejar 2002) facilitate TB-AIG item construction. The various studies differ in how item models have been specified and the role of transformer networks in the item generation process.

The most commonly used means to incorporate item models into TB-AIG is using a test item from an operational test as an item model and requesting the transformer network to construct isomorphic test items. This approach is reminiscent of early work within the item model approach. For instance, Shin and Gierl (2022) selected k = 5 passage-based reading comprehension items from an operationally used test in South Korea. Each item differed regarding the kind of inference required to solve the question following a short passage. The authors used ChatGPT with one-shot prompting to construct several isomorphic items for these k = 5 item models. The TB-AIG-constructed items were rated similar to the original items in terms of the perceived naturalness and attractiveness of the passage and in terms of the perceived attractiveness of the distractors by a panel of pre-service and in-service L2 English teachers. Similarly, Bezirhan and von Davier (2023) examined whether GPT-3 can be used to construct reading comprehension passages for informational and fictional text similar to the ones used in large-scale international studies. To this end, the authors used 1-shot prompting with or without additional specifications to construct isomorphic passages. They evaluated the similarity of the TB-AIG-constructed passages and the original ones in terms of their coherence, appropriateness, and readability for the target audience. The results indicated that providing additional information led to more isomorphic TB-AIG passages. However, the authors also noted that minor revisions and fact-checks were necessary during the passage generation process. Sayin and Gierl (2024) used a similar approach to enable GPT-3.5 to construct sentence comprehension items. This item type requires test-takers to mark one of five sentences that has a different topic than the other four sentences. The authors first designed a prompt for the four thematically matched sentences and requested the generation of k = 20 item isomorphs. The same procedure was used for the sentence with a different topic. The authors asked a panel of N = 3 subject-matter experts to evaluate the usability of TB-AIG items on a four-point single-item scale. All of the evaluated items were rated as either usable or requiring only minor revision. Sayin and Gierl (2024) administered k = 6 selected items in an operational reading comprehension test. Each of the k = 6 items was located at a fixed slot in the six booklets. They calculated classic item difficulty and item discrimination. Although item difficulty was in a narrow range, item discrimination for the test items hypothesized to have statistically identical item parameters varied considerably.

The second option to incorporate the item model approach into TB-AIG is to examine item design features of an existing operational test and to use cluster analysis to categorize existing items into clusters that subsequently serve as item models. This approach was used by Shin and Gierl (2022) to construct fill-in-the-blank reading comprehension items. The authors analyzed k = 2654 items from an operationally used test in South Korea in terms of their syntactic and semantic features. These data were used to distill k = 3 item models using hierarchical cluster analysis. Next, they analyzed k = 100 new human-made passages and categorized them into one of the three item model clusters. This information was used during prompting to determine the part of the text that should be blanked out and to construct the correct answer in addition to a set of distractors. The items were compared with the original human-made test items in terms of their syntactic and semantic item design features.

Kıyak and associates (Kıyak and Kononowicz 2025; Kıyak et al. 2024, 2025) suggested iteratively constructing item models based on a few sample items in the domain of clinical reasoning in medicine. The authors used GPT-Builder to construct a ChatGPT interface, which guides subject-matter experts through the process of constructing an item model commonly used in item model-based AIG. The process mirrors the one described by Gierl and Lai (2012). First, the expert enters a sample item. ChatGPT asks the subject-matter expert which part of the sample item should be varied and how the information in the variable parts relates to the distractors and the correct answer. Next, ChatGPT provides possible values for each variable element. The subject-matter expert can provide feedback on the factual correctness, which prompts ChatGPT to revise its original suggestions. Once the item model has been constructed, Kryak and associates propose implementing the newly constructed item model into an independent AIG software program, which can be used for item generation. The authors constructed various item models in this manner and generated k = 104 clinical reasoning test items. They randomly selected a set of k = 35 items and asked N = 18 experienced medical doctors to indicate the correct answer. Each item was reviewed by three medical doctors. Kıyak et al. (2025) report that 92.6 percent of all expert decisions corresponded with the scoring key proposed by ChatGPT.

As outlined above, most studies using *item model TB-AIG* utilized a weak theory approach and often merely resorted to judgements from subject-matter experts to validate the test items. Thus, important information on the dimensionality and psychometric characteristics of the TB-AIG items is missing. Furthermore, there seems to be a focus on the construction of item isomorphs (Bejar 2002) rather than item variants (Bejar 2002). This is in parts at odds with the demands of modern assessment practices, which require the construction of item variants and item isomorphs with either predictable or calibrated (=known) item parameters. Since descriptive and explanatory item response theory models were not fitted to the data, it is at present impossible to evaluate the cost savings that can be obtained with this approach during the item calibration phase and the item pool maintenance phase.

#### 4.3.2. Element-Based TB-AIG

Proponents of *element-based TB-AIG* manipulated radicals (Irvine 2002) independently of each other. Studies using element-based TB-AIG also often exhibit more variation in the extent to which radicals (Irvine 2002) were chosen on the basis of cognitive processing models.

In this section, we first outline research using a weak theory approach to element-based TB-AIG. For instance, Shultz et al. (2025) used ChatGPT with iterative prompt refinement to construct clinical reasoning items for a pharmacy exam. The authors entirely relied on subject-matter expertise during item construction with little to no reference to cognitive processing models that outline radicals and commonly held misconceptions in pharmacy (*weak theory approach*). Shultz et al. (2025) noted that about 63 percent of their automatically generated TB-AIG items passed the expert panel review because they either required no or only minor revision. Nonetheless, a notable number of these items had to be discarded in a subsequent calibration phase due to deficits in classical item discrimination (e.g., negative item discrimination). Similar results have been reported by Chauhan et al. (2025), who also resorted to a weak theory approach to element-based TB-AIG. The authors used iterative prompt engineering with explanations to construct clinical reasoning items in physiology using GPT-4 on an item-to-item basis. In line with the aforementioned study, the authors reported a notable item loss after the expert panel review for both TB-AIG items and test items written by human item writers. In addition, TB-AIG items exhibited lower item discrimination and were generally easier than test items written by human item writers. The lower discrimination was due to an increased number of non-functional distractors in the TB-AIG-generated test items. Similarly, Lin and Chen (2024) used iterative zero-shot prompting with ChatGPT to construct passage-based reading comprehension items for two passages taken from an operational high-stakes test in China. They administered k = 18 TB-AIG items together with the original k = 10 items constructed for these two passages by human item writers. The authors report an item loss of ≈26% of the TB-AIG test items due to a misfit of the 2PL model. However, the distribution of the 2PL item parameters of the remaining TB-AIG items was similar to items made by human item writers. In addition, the quality of the remaining TB-AIG items was also judged to be similar to the items made by human item writers in a human subject-matter review process. The authors also examined the excluded TB-AIG items and noticed issues in the TB-AIG-constructed distractors as a possible reason for the misfit. Zu et al. (2023) used fine-tuning and iterative prompt engineering with GPT-2 to construct distractors for an operationally used fill-in-the-blank vocabulary test. They also examined different prompt designs and compared their results in terms of relevant linguistic features to a rule-based distractor construction method and to distractors constructed by human item writers. Their results indicated that more fine-tuned prompts yielded distractors most similar to human item writers.

By contrast, other studies resorted to existing cognitive processing models in selecting the radicals (*strong theory approach*). For instance, Attali et al. (2022) used iterative few-shot prompts with GPT-3 to construct k = 789 passages and test items measuring predefined levels of reading comprehension. That is, the authors analyzed the initial output of GPT-3 in terms of a set of radicals and refined their prompts to command GPT-3 to revise the initial test items according to their specification. This process was repeated until a human subject-matter expert was content with the result. Next, the TB-AIG items were reviewed by a panel of subject-matter experts. Attali et al. (2022) report that they were able to retain k = 454 (57.54%) items after the expert panel review. TB-AIG items that passed the panel review were administered to a few thousand test-takers. Each test-taker only worked on one of the TB-AIG items, which was administered as the last item in an operational high-stakes practice test. Although the authors were unable to examine the fit of descriptive item response theory models (De Boeck and Wilson 2004), their research design at least enabled them to calculate classic item parameters such as item difficulty and discrimination. Both classic test theoretical item statistics varied considerably. Runge et al. (2024) also used iterative prompt refinement to generate listening comprehension scripts and multiple-choice test items. The authors report that 719 (99%) of the TB-AIG test items passed the final expert panel review and were therefore subjected to the item calibration phase. Test-takers worked on two TB-AIG items administered at the end of an operational practice test. Classic test theoretical statistics (here, item difficulty and discrimination) were calculated. Runge et al. (2024) also tried to predict the item parameters on the basis of a set of radicals using multiple regression analysis. The radicals jointly accounted for R^2^ = 0.10 (item discrimination) to R^2^ = 29 (item difficulty) of the variance in the item parameters. Last, but not least, Schroeders and Achaa-Amankwaa (2025) used a combination of human item writing and element-based TB-AIG to construct a vocabulary item pool (k = 110), which required test-takers to identify an actual German word presented together with four pseudo-words. Using an incomplete link design, the item pool was calibrated by means of the 2PL model. In addition, multiple logistic regression analysis was used to predict the item difficulty parameter. The systematically manipulated radicals accounted for 57.4% of the variance in the 2PL item difficulty parameters. Similar to the aforementioned studies, the authors noted that severe prompt tuning and subject-matter reviews were required to get ChatGPT to construct the test items.

Research on *element-based TB-AIG* seems to resort to item calibration and explanatory item response theory modeling more frequently than item model-based TB-AIG. In addition, the authors resort more often to theoretical models to select radicals (Irvine 2002). However, there seems to be considerable variation in the extent to which this is the case. Furthermore, even strong(er)-theory element-based TB-AIG utilized cognitive processing models and/or computational models to a lesser extent than either the cognitive design system approach or the automatic min-max approach. This is unfortunate, since a more theory-based selection of radicals (Irvine 2002) seems to lead to a higher correlation between empirically estimated and predicted item parameters even in the verbal ability domain (cf. Arendasy et al. 2020; Kapoor et al. 2025). Thus, a stronger theoretical focus of the TB-AIG item construction process may be a fruitful area of further research. This may also include further refining the prompts to incorporate sketches, for example, story plots—like the ones commonly used by professional writers and game developers—to systematically manipulate reading comprehension inference demands. Recent research in computer science indicates that such an approach is in principle technically feasible (cf. Wang et al. 2024). Last, but not least, a stronger focus on descriptive and explanatory item response theory modeling (cf. De Boeck and Wilson 2004) and exploring the feasibility of including ideas similar to a quality control component would be desirable to build even stronger validity arguments for TB-AIG-constructed test items.

## 5. Discussion

The initial phase of switching from fixed-item linear paper-pencil tests to CATs and MSTs was associated with the hope that this would lead to a more flexible diagnostic process in addition to a reduction in costs (Luecht 2005). However, the hoped-for cost reduction has not been fulfilled. Practitioners and researchers soon realized that testing on demand, providing multiple retest opportunities, and equilizing individual differences in test preparation by means of providing cost-free practice-based training come at a cost. To ensure that these practices do not compromise the psychometric characteristics of the cognitive ability test, test developers need to be able to construct large pools of test items that have to be constantly monitored and updated (e.g., Ariel et al. 2006; Lang 2011; Liu et al. 2019; Segall 2004; Sinharay 2017; van der Linden and Glas 2010). Human item writing thus soon turned out to be a critical bottleneck in psychological and educational assessment (cf. Drasgow et al. 2006; Hornke and Habon 1986; Kosh et al. 2019; Luecht 2005; Wainer 2002; Zwick 2002) and has often been blamed for the fact that the hoped-for cost reductions were not achieved (cf. Luecht 2005).

### 5.1. Capacity of Item Generations Methods to Deal with the Specific Item Construction Demands

Research (e.g., He and Reckase 2014; Reckase 2010; Reckase et al. 2019; Veldkamp and van der Linden 2010) indicates that the distribution of the item parameters of an item pool should mimic the distribution of the person parameters of the target population. Furthermore, the item pool should only contain item variants (Bejar 2002). This is because item isomorphs (Bejar 2002) may introduce local dependencies, which lead to biased person parameter estimates (cf. Segall 2004; Glas and van der Linden 2003). To support item- and content-exposure control (cf. Georgiadou et al. 2007; Gierl et al. 2022a; Lim and Choi 2024), each item pool should contain a fair amount of *psychometrically matched instances* (Arendasy and Sommer 2013a) that are evenly distributed across the entire continuum of the latent trait. Psychometrically matched instances are test items that are (almost) identical to another test item in terms of their item parameters despite exhibiting a different combination of radicals. Despite the success of this approach in preventing item disclosure and reducing retest effects (e.g., Guo et al. 2009; Arendasy and Sommer 2017), the item construction demands are rather severe. To determine the extent to which the various item construction methods are able to keep pace with these demands, we need to determine how many different item variants and psychometrically matched instances each method is able to produce.

In the *item model approach* (e.g., Bejar 2002; Choi and Zhang 2019; Gierl et al. 2022b; LaDuca et al. 1986) and the *cognitive design model approach* (cf. Embretson 1998, 2002, 2016; Embretson and Yang 2007; Gorin 2006), the number of item variants is restricted by the number of item models. As outlined in Section 3.1 and Section 3.2, the number of item models varies considerably, but rarely exceeds 60 item models. Taking into account that, based on their expected item family function, some item models were intended to construct item variants, we would obtain an upper bound of ≤180 item variants in most practical applications. Unfortunately, there is to date little information on the extent to which these two approaches are capable of generating psychometrically matched item instances along the entire latent trait continuum. Given its importance to support the use of item- and content-exposure control algorithms, and to continuously update an existing item pool, this would be a fruitful field for further scientific research. By contrast, research using the *automatic min-max approach* (cf. Arendasy 2004; Arendasy and Sommer 2011, 2012a; Arendasy et al. 2020; Arendasy et al. 2024) often reports a number of item variants ranging from k = 120 to k = 320. However, the authors stress that these numbers may not be exhaustive but represent a rather representative selection of the number of item variants that can be generated. In addition, Arendasy and Sommer (2013a) report that most item generators were able to construct psychometrically matched instances. However, the number of psychometrically matched instances varied along various points of the latent trait continuum. Regarding TB-AIG, the low number of empirical studies using item calibration to evaluate the TB-AIG items prevents an evaluation of the extent to which this item construction method is able to meet the rather specific item demands in constructing and maintaining an initial item pool. As outlined in Section 4.3, more research on the psychometric characteristics of TB-AIG test items is needed before their contribution can be evaluated.

In addition to item variants (Bejar 2002), item isomorphs (Bejar 2002) also contribute to increasing test security. More specifically, item isomorphs could be used to construct multiple rotating item pools, which have been shown to reduce item exposure and enhance test security (cf. Ariel et al. 2006; Zhang et al. 2012). The various approaches to automatic item generation (AIG) do not seem to differ much in the extent to which they enable the construction of item isomorphs. However, the practical implementation of constructing item isomorphs seems to be more convenient in the case of the item model approach compared to the element-based approach. Tentative evidence indicates that TB-AIG also seems to be capable of constructing item isomorphs. However, more studies are needed in which TB-AIG items are calibrated to determine the extent to which isomorphic test items indeed exhibit (almost) statistically identical item parameters (cf. Section 4.3).

### 5.2. Capacity of Item Generation Methods to Provide Cost-Free Practice-Based Training

Based on evidence that both practice-based training and test coaching have been shown to be effective at increasing test-takers’ test scores, several researchers (e.g., AERA et al. 2018; Arendasy et al. 2016; Campion et al. 2019; Hermes et al. 2019; Lee et al. 2023a; Sommer et al. 2025) proposed providing cost-free practice-based training in order to equalize individual differences in access to commercial test preparation methods. The standards for educational and psychological assessment (AERA et al. 2018) propose that sample-test items used in practice-based training and test coaching should be representative of the actual test items. However, the practice-based training items should not be identical to operationally used test items in terms of their radical combinations. Furthermore, if sample-test items include feedback and tutorials, they should cover all difficulty levels of the operationally used test items (cf. Arendasy et al. 2016; Krautter et al. 2021; Sommer et al. 2025). However, providing effective feedback requires that test-takers’ cognitive processes and misconceptions are sufficiently well understood, and that reasons for incorrect responses can be inferred from their item responses. Thus, test item construction and sample-test item construction should ideally be based on validated cognitive processing models developed by cognitive scientists and professionals working in the domain of subject-matter didactics. In sum, the above-mentioned requirements for the construction of sample-test items used in practice-based training imply that sample-test item pools should ideally consist of item variants constructed by means of strong(er) theory. The main argument against the use of item isomorphs in practice-based training is that item isomorphs have sometimes been shown to lead to local dependencies between isomorphic items, and more pronounced score gains (cf. Arendasy and Sommer 2013a, 2017; Matton et al. 2011; Morley et al. 2004). Both effects induce a bias in the person parameter estimates, which calls the validity and fairness of the psychometric test into question. Since shorter time intervals between practice-based training and test-taking are common in high-stakes test preparation (cf. Powers 2012), the use of item isomorphs should be avoided. Instead, a separate item pool consisting of item variants (Bejar 2002) and psychometrically matched intances (Arendasy and Sommer 2013a) should be constructed for use in cost-free practice-based training. Unfortunately, this extends the item construction demands beyond the initial construction and maintenance of multiple item pool used for assessment proposes to the simultaneous construction of item pools used for assessment and an additional item pool used in practice-based training (cf. Arendasy et al. 2016; Hermes et al. 2019; Lee et al. 2023a; Sommer et al. 2025). Given that the specifications based on the empirical evidence outlined in Section 1.3 are identical to those outlined in Section 5.1, the evaluation of the extent to which current methods of automatic item generation (AIG) and transformer-based automatic item generation (TB-AIG) are able to meet these requirements is also identical.

### 5.3. Potential Cost Savings During the Actual Item Construction Phase

Despite differences in the various methods of automatic item generation (AIG)—including transformer-based automatic item generation (TB-AIG)—in terms of how radicals are deduced and manipulated (e.g., weak vs. strong theory approach, schema-based vs. element-based approach), all methods aim to make the item construction process more scalable and cost-efficient. Cost savings during the item construction process itself heavily depend on the degree of automatization that is possible. In our discussion of TB-AIG, we outlined the reasons for the recommendations to keep humans in the loop during the entire item construction process (cf. Attali et al. 2022; Bulut et al. 2024; Hao et al. 2024; von Davier 2019). This implies that semi-automatic TB-AIG requires more resources in terms of time and human manpower compared to fully automatic item generation. Thus, the cost saving in fully automatic AIG is naturally higher than in TB-AIG and semi-automatic AIG. Nevertheless, both semi-automatic item generation and TB-AIG seem to outperform human item writing in terms of the time needed to construct the test items, even when considering that 1 to 43 percent of the TB-AIG items were discarded after the final expert panel review (cf. Attali et al. 2022; Runge et al. 2024).

### 5.4. Potential Cost Savings in the Item Calibration Phase and the Item Pool Maintenance Phase

The various methods of automatic item generation (AIG) and transformer-based automatic item generation (TB-AIG) also differ in the extent to which they lead to cost savings in the item calibration phase and the item pool maintenance phase. Most studies conducted within the *item model approach* (e.g., Bejar 2002; Choi and Zhang 2019; Gierl et al. 2022b; LaDuca et al. 1986) and the *cognitive design model approach* (cf. Embretson 1998, 2002, 2016; Embretson and Yang 2007; Gorin 2006) utilizes the 2PL model to calibrate the test items. This also holds for the few studies using TB-AIG that use item response theory to calibrate the semi-automatically constructed TB-AIG items (e.g., Lin and Chen 2024; Schroeders and Achaa-Amankwaa 2025). By contrast, proponents of the automatic min-max approach (cf. Arendasy and Sommer 2011, 2012a; Arendasy et al. 2024) utilized the 1PL Rasch model for item calibration (cf. Section 3.3). These two descriptive item response theory models differ in the number of item parameters that need to be estimated. While the 2PL model estimates an item difficulty parameter and an item discrimination parameter for each individual test item, the 1PL Rasch model restricts the item discrimination parameters to be equal across all test items (assumption of essential tau equivalent measurement). As a consequence, the item discrimination parameters do not need to be estimated. Several simulation studies (e.g., Belzak 2019; Sahin and Anil 2017; Schroeders and Gnambs 2025; Zimmer et al. 2024; Zwick et al. 1995) indicate that—all other things being equal—the 2PL Rasch model requires larger sample sizes to obtain reliable item parameter estimates and to conduct DIF analyses, than the 1PL Rasch model. Given that sample size is a major cost factor, cost savings may be achieved by automatically generating test items whose data have a higher chance to fit the 1PL Rasch model no worse than the 2PL model without running the risk of construct underrepresentation. To this end, Arendasy and associates (cf. Arendasy and Sommer 2011, 2012a; Arendasy et al. 2024 ; Hines 2017) proposed conducting psychometric experiments to examine possible reasons for item-specific differences in the empirically estimated item discrimination parameters and to evaluate whether these reasons are linked to cognitive processes deemed to be central to the latent cognitive ability trait (for examples, see Arendasy and Sommer 2005, 2010, 2012b, 2013b; Arendasy et al. 2012).

A similar argument can be made with regard to cost savings in DIF analyses. Several studies (e.g., Kara and Dogan 2022; Sahin Kursad and Yalcin 2024; Piromsombat 2014) indicate that ignoring DIF in CAT and MST leads to biased person parameter estimates. Thus, DIF analyses should be mandatory. The various approaches to automatic item generation (AIG)—including transformer-based automatic item generation (TB-AIG)—also differ regarding the way in which DIF items and items exhibiting a misfit to the chosen descriptive item response theory model (here: 1PL model vs. 2PL model) are handled. Within the *item model approach* (e.g., Bejar 2002; Choi and Zhang 2019; Gierl et al. 2022b; LaDuca et al. 1986), the *cognitive design model approach* (cf. Embretson 1998, 2002, 2016; Embretson and Yang 2007; Gorin 2006), and TB-AIG (e.g., Lin and Chen 2024; Schroeders and Achaa-Amankwaa 2025), items are often excluded based on technical item selection criteria. This has been reported to lead to an item loss of ≤30 percent during the item calibration phase. By contrast, the automatic min-max approach proposes to either model sources of the misfit and/or DIF in terms of radicals or functional constraints (e.g., Arendasy and Sommer 2013b; Rajeb et al. 2024; Yu 1994), or to conduct psychometric experiments with precise hypotheses on possible causes for an item misfit or DIF deduced from cognitive processing models (cf. Arendasy and Sommer 2005, 2010, 2012b, 2013b; Arendasy et al. 2012; Hines 2017). The results are subsequently used in the construction and evaluation of the quality control component, which aims to minimize the likelihood of DIF due to construct-unrelated cognitive processes. This process has been reported to lead to an item loss of ≤1 percent (for a replication of this finding, see Arendasy et al. 2020; Hines 2017). This indicates that resorting to a strong(er) theory approach may not only lead to an improvement in validity but also to cost savings during the calibration of the initial item pool.

In addition, automatic item generation (AIG) also enables cost savings during the item pool maintenance phase. While traditional human item writing requires that all items need to be calibrated individually, AIG proposes either to calibrate item models or individual radicals as the basic building blocks of the item generators (cf. Section 3.1, Section 3.2 and Section 3.3). Since most studies in TB-AIG did not model the item parameters in terms of their radicals (for an exception, see Runge et al. 2024; Schroeders and Achaa-Amankwaa 2025), the extent to which TB-AIG may lead to cost savings in the item pool maintenance phase remains unclear. Several simulation studies (e.g., Bejar et al. 2002; Colvin et al. 2016; Glas and van der Linden 2003; Someshwar 2024; Tian and Choi 2023) indicate that—provided that predicted and calibrated item parameters are highly correlated (R ≥ 0.90)—using expected item family functions instead of the actually calibrated test item parameters is feasible and only leads to a negligible bias in the person parameter estimates that can be offset by a slight increase in the number of items administered during CAT. Almost identical results have been reported for the use of item parameters predicted on the basis of the radicals themselves (e.g., Doebler 2012; Embretson 1999; Freund et al. 2008; Mislevy et al. 1993; Matteucci et al. 2012; Someshwar 2024). Provided that studies using the *cognitive design system approach* (R = 0.70 to R = 0.90) and the *automatic min-max approach* (R = 0.89 to R = 0.96) often yielded a high correlation between predicted and empirically estimated item parameters, the results indicate that cost savings in the item pool maintenance phase may be possible by skipping the item calibration phase and using predicted item parameters instead of actually calibrated item parameters (for details, see Section 3.2 and Section 3.3). Interestingly, strong(er) theory item model approaches, which aim to model the variance of the item parameters within an item model in terms of radicals (cf. Cho et al. 2014; Geerlings et al. 2013; Glas et al. 2016), tend to perform better in this regard than weak(er) theory item model approaches. Thus, resorting to cognitive processing models and computational models in choosing radicals (Irvine 2002) not only provides valuable validity evidence in terms of cognitive processes involved in test item solving (for a review, see AERA et al. 2018) but also leads to cost savings in the item pool maintenance phase. The beneficial effect of strong(er)-theory approaches is also evident in human item writing (cf. Section 2). The more human item writers know about construct-related cognitive processes and how they are triggered by certain item design features, the better they are able to tailor their item writing to the demands of the specifications of the blueprint for an ideal item pool. This recurring finding across different item construction methods points to the potential benefit of a closer cooperation between theoretical psychologists, who are concerned with the development and evaluation of cognitive processing models and computational models, and applied psychometricians, who aim to develop valid and fair cognitive ability tests (for a similar argument, see Smaldino 2020).

### 5.5. Differences in Test Security Concerns

Without a doubt, the item model approach (e.g., Bejar 2002; Choi and Zhang 2019; LaDuca et al. 1986) is the most popular approach to AIG to date. This has led some researchers (e.g., Circi et al. 2023; Gierl et al. 2022a; von Davier 2018) to incorrectly equate the item model approach with the entire field of AIG. The main reason for this is that item models can be constructed using both weak and strong theoretical foundations (Drasgow et al. 2006). In addition, item models are convenient to implement in programming terms (Embretson and Yang 2007). Furthermore, usability studies indicate that item models are easier to handle for subject-matter experts, who often need to review and revise the item models without having a background in programming (cf. Falcão et al. 2023, 2024; Choi and Zhang 2019; Gierl and Lai 2012; Lai et al. 2016). The ease in handling item models—also partly due to their often hierarchical structure and finite number of item models—surely contributes to the cost savings reported during the actual item construction process and the development of the item generator (cf. Falcão et al. 2023, 2024; Choi and Zhang 2019; Gierl and Lai 2012; Gierl et al. 2022a). However, these benefits also come at a cost. Using item models in CAT and MST requires both content coding and item exposure control at the item- and item model level (cf. Gierl et al. 2022a). This is necessary to ensure that isomorphic test items (Bejar 2002) are not administered within the same test session to prevent a bias in the person parameter estimates (cf. Glas and van der Linden 2003; Segall 2004). Furthermore, the ease of use and the often hierarchical structure of item models make them more vulnerable to organized item theft. Research (cf. Yi et al. 2008) indicates that item theft detrimentally affects the person parameter estimates and, consequentially, the validity and fairness of the psychometric test. If an entire item model has been disclosed or leaked, all items generated by that item model are compromised and need to be removed from operational use. By contrast, in the element-based approach, radicals (Irvine 2002) are manipulated independently of each other. While this may complicate the ease of use of the item generator by non-experts, it also enhances test security. This is because item theft can only occur on an item-to-item basis instead of at the level of item models or item model families. Thus, using item models requires additional technical test- or item-model security measures to protect the item models from item disclosure and organized item theft (for an overview, see Foster 2016). A related argument can be made with regard to the use of open and commercial transformer models in item generation (cf. Bulut et al. 2024; Hao et al. 2024; Section 4.2).

## 6. Concluding Remarks

Thus, different methods of automatic item generation (AIG; Arendasy 2004; Arendasy and Sommer 2011, 2012a; Arendasy et al. 2024; Embretson and Yang 2007; Irvine and Kyllonen 2002), including transformer-based automatic item generation (TB-AIG: Attali et al. 2022; von Davier 2019), come with unique costs and benefits at various stages of the construction and maintenance phase of multiple item pools needed in assessment as well as cost-free practice-based training. Unfortunately, current cost-benefit studies neither take differences between methods in the costs associated with the construction and maintenance of multiple item pools into account nor do they factor in differences in the costs associated with varying levels of risk of item disclosure and organized item theft (cf. Kosh et al. 2019). An update of such cost-benefit analyses that takes these aspects into account would help test developers choose a particular approach to automatic (including transformer-based automatic) item generation that is more tailored to their current situational constraints and their short-term and long-term needs.

## Abbreviations

The following abbreviations are used in this manuscript:

AIG	Automatic item generation
CAT	Computerized adaptive test
DIF	Differential item functioning
LLTM	Linear Logistic Test Model
LOFT	Linear on-the-fly test
MST	Multi-stage test
TB-AIG	Transformer-based automatic item generation

## References

[B1-jintelligence-13-00102] Abu-Haifa Mohammad, Etawi Bara’a, Alkhatatbeh Huthaifa, Ababneh Ayman (2024). Comparative analysis of ChatGPT, GPT-4, and Microsoft Copilot Chatbots for GRE test. International Journal of Learning, Teaching and Educational Research.

[B2-jintelligence-13-00102] Ahn Jihyun J., Yin Wenpeng (2025). Prompt-Reverse Inconsistency: LLM Self-Inconsistency Beyond Generative Randomness and Prompt Paraphrasing. arXiv.

[B3-jintelligence-13-00102] Al Faraby Said, Romadhony Ade, Adiwijaya (2024). Analysis of llms for educational question classification and generation. Computers and Education: Artificial Intelligence.

[B4-jintelligence-13-00102] Allalouf Avi, Ben-Shakhar Gershon (1998). The effect of coaching on the predictive validity of scholastic aptitude tests. Journal of Educational Measurement.

[B5-jintelligence-13-00102] American Educational Research Association (AERA), American Psychological Association (APA), National Council on Measurement in Education (NCME) (2018). Standards for Educational and Psychological Testing.

[B6-jintelligence-13-00102] Anderson John R., Fincham Jon M., Douglass Scott (1997). The role of examples and rules in the acquisition of a cognitive skill. Journal of Experimental Psychology: Learning, Memory and Cognition.

[B7-jintelligence-13-00102] Appelhaus Stefan, Werner Susanne, Grosse Pascal, Kämmer Juliane E. (2023). Feedback, fairness, and validity: Effects of disclosing and reusing multiple-choice questions in medical schools. Medical Education Online.

[B8-jintelligence-13-00102] Appelrouth Jed I., Zabrucky Karen M., Moore DeWayne (2017). Preparing students for college admissions tests. Assessment in Education: Principles, Policy and Practice.

[B9-jintelligence-13-00102] Arendasy Martin (2000). Psychometrischer Vergleich Computergestützter Vorgabeformen bei Raumvorstellungsaufgaben: Stereoskopisch-Dreidimensionale und Herkömmlich-Zweidimensionale Darbietung. Ph.D thesis.

[B10-jintelligence-13-00102] Arendasy Martin (2004). Automatisierte Itemgenerierung und Psychometrische Qualitätssicherung am Beispiel des Matrizentests GEOM.

[B11-jintelligence-13-00102] Arendasy Martin, Sommer Markus (2005). The effect of different types of perceptual manipulations on the dimensionality of automatically generated figural matrices. Intelligence.

[B12-jintelligence-13-00102] Arendasy Martin, Sommer Markus (2007). Using psychometric technology in educational assessment: The case of a schema-based isomorphic approach to the automatic generation of quantitative reasoning items. Learning and Individual Differences.

[B13-jintelligence-13-00102] Arendasy Martin, Sommer Markus (2010). Evaluating the contribution of different item features to the effect size of the gender difference in three-dimensional mental rotation using automatic item generation. Intelligence.

[B14-jintelligence-13-00102] Arendasy Martin, Sommer Markus, Hornke Lutz F., Amelang Manfred, Kersting Martin (2011). Automatisierte Itemgenerierung: Aktuelle Ansätze, Anwendungen und Forschungen. Enzyklopädie für Psychologie: Methoden der Psychologischen Diagnostik.

[B15-jintelligence-13-00102] Arendasy Martin, Sommer Markus (2012a). Using automatic item generation to meet the increasing item demands of high-stakes assessment. Learning and Individual Differences.

[B16-jintelligence-13-00102] Arendasy Martin, Sommer Markus (2012b). Gender differences in figural matrices: The moderating role of item design features. Intelligence.

[B17-jintelligence-13-00102] Arendasy Martin, Sommer Markus (2013a). Quantitative differences in retest effects across different methods used to construct alternate test forms. Intelligence.

[B18-jintelligence-13-00102] Arendasy Martin, Sommer Markus (2013b). Reducing response elimination strategies enhances the construct validity of figural matrices. Intelligence.

[B19-jintelligence-13-00102] Arendasy Martin, Sommer Markus, Hergovich Andreas (2007). Psychometrische Technologie: Automatische Zwei-Komponenten-Itemgenerierung am Beispiel eines neuen Aufgabentyps zur Messung der Numerischen Flexibilität. Diagnostica.

[B20-jintelligence-13-00102] Arendasy Martin, Sommer Markus, Mayr Friedrich (2012). Using automatic item generation to simultaneously con-struct German and English versions of a verbal fluency test. Journal of Cross-Cultural Psychology.

[B21-jintelligence-13-00102] Arendasy Martin, Sommer Markus, Gittler Georg (2010). Combining automatic item generation and experimental designs to investigate the contribution of cognitive components to the gender difference in mental rotation. Intelligence.

[B22-jintelligence-13-00102] Arendasy Martin, Sommer Markus, Gittler Georg (2020). Manual Intelligence-Struktur-Battery 2 (INSBAT-2).

[B23-jintelligence-13-00102] Arendasy Martin, Sommer Markus, Hergovich Andreas, Feldhammer Martina (2011). Evaluating the impact of depth cue salience in working three-dimensional mental rotation tasks by means of psychometric experiments. Learning and Individual Differences.

[B24-jintelligence-13-00102] Arendasy Martin, Sommer Markus, Gittler Georg, Hergovich Andreas (2006). Automatic generation of quantitative reasoning items: Pilot study. Journal of Individual Differences.

[B25-jintelligence-13-00102] Arendasy Martin E., Sommer Markus (2017). Reducing the effect size of the retest effect: Examining different approaches. Intelligence.

[B26-jintelligence-13-00102] Arendasy Martin E., Sommer Markus, Gutierrez-Lobos Karin, Punter Joachim F. (2016). Do individual differences in test preparation compromise the measurement fairness of admission tests?. Intelligence.

[B27-jintelligence-13-00102] Arendasy Martin E., Sommer Markus, Tschiesner Reinhard, Feldhammer-Kahr Martina, Umdasch Konstantin (2024). Using automatic item generation to construct scheduling problems measuring planning ability. Intelligence.

[B28-jintelligence-13-00102] Ariel Adelaide, van Der Linden Wim J., Veldkamp Bernard P. (2006). A strategy for optimizing item-pool management. Journal of Educational Measurement.

[B29-jintelligence-13-00102] Attali Yigal, Runge Andrew, LaFlair Geoffrey T., Yancey Kevin, Goodwin Sarah, Park Yena, von Davier Alina A. (2022). The interactive reading task: Transformer-based automatic item generation. Frontiers in Artificial Intelligence.

[B30-jintelligence-13-00102] Attali Yigal, Saldivia Luis, Jackson Carol, Schuppan Fred, Wanamaker Wilbur (2014). Estimating Item Difficulty with Comparative Judgments.

[B31-jintelligence-13-00102] Baldonado Angela A., Svetina Dubravka, Gorin Joanna (2015). Using necessary information to identify item dependence in passage-based reading comprehension tests. Applied Measurement in Education.

[B32-jintelligence-13-00102] Balestri Roberto (2025). Gender and content bias in Large Language Models: A case study on Google Gemini 2.0 Flash Experimental. Frontiers in Artificial Intelligence.

[B33-jintelligence-13-00102] Bangert-Drowns Robert L., Kulik James A., Kuli Chen-Lin C. (1983). Effects of coaching programs on achievement test performance. Review of Educational Research.

[B34-jintelligence-13-00102] Becker Betsy J. (1990). Coaching for the Scholastic Aptitude Test: Further synthesis and appraisal. Review of Educational Research.

[B35-jintelligence-13-00102] Beg Mirza A., Tabassum Afifa, Ali Sobia (2021). Role of faculty development workshop for improving MCQS quality in basic medical sciences. Biomedica.

[B36-jintelligence-13-00102] Bejar Isaac I. (1983). Subject matter experts’ assessment of item statistics. Applied Psychological Measurement.

[B37-jintelligence-13-00102] Bejar Isaac I., Irvine Sidney H., Kyllonen Patrick C. (2002). Generative testing: From conception to implementation. Item Generation for Test Development.

[B38-jintelligence-13-00102] Bejar Isaac I., Lawless René R., Morley Mary E., Wagner Michael E., Bennett Randy E., Revuelta Javier (2002). A Feasibility Study of On-the-Fly Item Generation in Adaptive Testing (GRE Board Professional Rep. No. 98-12P).

[B39-jintelligence-13-00102] Bejar Isaac I., Chaffin Roger, Embretson Susan (2012). Cognitive and Psychometric Analysis of Analogical Problem Solving.

[B40-jintelligence-13-00102] Belzak William C., Naismith Ben, Burstein Jill (2023). Ensuring fairness of human-and AI-generated test items. International Conference on Artificial Intelligence in Education.

[B41-jintelligence-13-00102] Belzak William C. M. (2019). Testing differential item functioning in small samples. Multivariate Behavioral Research.

[B42-jintelligence-13-00102] Berenbon Rebecca F., McHugh Bridget C. (2023). Do subject matter experts’ judgments of multiple-choice format suitability predict item quality?. Educational Measurement: Issues and Practice.

[B43-jintelligence-13-00102] Bethell-Fox Charles E., Lohman David F., Snow Richard E. (1984). Adaptive reasoning: Componential and eye movement analysis of geometric analogy performance. Intelligence.

[B44-jintelligence-13-00102] Bezirhan Ummugul, von Davier Matthias (2023). Automated reading passage generation with OpenAI’s large language model. Computers and Education: Artificial Intelligence.

[B45-jintelligence-13-00102] Bhayana Rajesh, Krishna Satheesh, Bleakney Robert R. (2023). Performance of ChatGPT on a radiology board-style examination: Insights into current strengths and limitations. Radiology.

[B46-jintelligence-13-00102] Blum Diego, Holling Heinz (2018). Automatic generation of figural analogies with the imak package. Frontiers in Psychology.

[B47-jintelligence-13-00102] Borsboom Denny, Romeijn Jan-Willem, Wicherts Jelte M. (2008). Measurement invariance versus selection invariance: Is fair selection possible?. Psychological Methods.

[B48-jintelligence-13-00102] Bozkurt Aras, Sharma Ramesh C. (2023). Generative AI and prompt engineering: The art of whispering to let the genie out of the algorithmic world. Asian Journal of Distance Education.

[B49-jintelligence-13-00102] Briggs Derek C. (2009). Preparation for College Admission Exams (2009 NACAC Discussion Paper).

[B50-jintelligence-13-00102] Brown Tom B., Mann Benjamin, Ryder Nick, Subbiah Melanie, Kaplan Jared, Dhariwal Prafulla, Neelakantan Arvind, Shyam Pranav, Sastry Girish, Askell Amanda (2020). Language models are few-shot learners. Advances in Neural Information Processing Systems.

[B51-jintelligence-13-00102] Buchmann Claudia, Condron Dennis J., Roscigno Vincent J. (2010). Shadow education, American style: Test preparation, the SAT and college enrollment. Social Forces.

[B52-jintelligence-13-00102] Bulut Okan, Beiting-Parrish Maggie, Casabianca Jodi M., Slater Sharon C., Jiao Hong, Song Dan, Morilova Polina (2024). The rise of artificial intelligence in educational measurement: Opportunities and ethical challenges. arXiv.

[B53-jintelligence-13-00102] Burke Eugene F. (1997). A short note on the persistence of retest effects on aptitude scores. Journal of Occupational and Organizational Psychology.

[B54-jintelligence-13-00102] Burns Gary N., Siers Brian P., Christiansen Neil D. (2008). Effects of providing pre-test information and preparation materials on applicant reactions to selection procedures. International Journal of Selection and Assessment.

[B55-jintelligence-13-00102] Calamia Matthew, Markon Kristian, Tranel Daniel (2012). Scoring higher the second time around: Meta-analyses of practice effects in neuropsychological assessment. The Clinical Neuropsychologist.

[B56-jintelligence-13-00102] Campion Michael C., Campion Emily D., Campion Michael A. (2019). Using practice employment tests to improve recruitment and personnel selection outcomes for organizations and job seekers. Journal of Applied Psychology.

[B57-jintelligence-13-00102] Chan Kuang W., Ali Farhan, Park Joonhyeong, Sham Kah S. B., Tan Erdalyn Y. T., Chong Francis W. C., Sze Guan K. (2025). Automatic item generation in various STEM subjects using large language model prompting. Computers and Education: Artificial Intelligence.

[B58-jintelligence-13-00102] Chauhan Archana, Khaliq Farah, Nayak Kirtana Raqhurama (2025). Assessing quality of scenario-based multiple-choice questions in physiology: Faculty-generated vs. ChatGPT-generated questions among phase I medical students. International Journal of Artificial Intelligence in Education.

[B59-jintelligence-13-00102] Cho Sun-Joo, De Boeck Paul, Embretson Susan, Rabe-Hesketh Sophia (2014). Additive multilevel item structure models with random residuals: Item modeling for explanation and item generation. Psychometrika.

[B60-jintelligence-13-00102] Choi Jaehwa, Zhang Xinxin (2019). Computerized item modeling practices using computer adaptive formative assessment automatic item generation system: A tutorial. The Quantitative Methods for Psychology.

[B61-jintelligence-13-00102] Chung Jinmin, Kim Sungyeun (2024). Comparison of rule-based models and Large Language Models in item and feedback generation. Journal of Science Education.

[B62-jintelligence-13-00102] Circi Ruhan, Hicks Juanita, Sikali Emmanuel (2023). Automatic item generation: Foundations and machine learning-based approaches for assessments. Frontiers in Education.

[B63-jintelligence-13-00102] Colvin Kimberly F., Keller Lisa A., Robin Frederic (2016). Effect of imprecise parameter estimation on ability estimation in a multistage test in an automatic item generation context. Journal of Computerized Adaptive Testing.

[B64-jintelligence-13-00102] Daniel Robert C., Embretson Susan E. (2010). Designing cognitive complexity in mathematical problem-solving items. Applied Psychological Measurement.

[B65-jintelligence-13-00102] De Boeck Paul, Wilson Mark (2004). Explanatory Item Response Models: A Generalized Linear and Nonlinear Approach.

[B66-jintelligence-13-00102] Denker Marek, Schütte Clara, Kersting Martin, Weppert Daniel, Stegt Stephan J. (2023). How can applicants’ reactions to scholastic aptitude tests be improved? A closer look at specific and general tests. Frontiers in Education.

[B67-jintelligence-13-00102] Devlin Jacob, Chang Ming-Wei, Lee Kenton, Toutanova Kristina (2018). Bert: Pre-training of deep bidirectional transformers for language understanding. arXiv.

[B68-jintelligence-13-00102] Doebler Anna (2012). The problem of bias in person parameter estimation in adaptive testing. Applied Psychological Measurement.

[B69-jintelligence-13-00102] Doebler Anna, Holling Heinz (2016). A processing speed test based on rule-based item generation: An analysis with the Rasch Poisson Counts model. Learning and Individual Differences.

[B70-jintelligence-13-00102] Draheim Christopher, Harrison Tyler L., Embretson Susan E., Engle Randall W. (2018). What item response theory can tell us about the complex span tasks. Psychological Assessment.

[B71-jintelligence-13-00102] Drasgow Fritz, Luecht Richard M., Bennett Randy, Brennan R. L. (2006). Technology and testing. Educational Measurement.

[B73-jintelligence-13-00102] Eleragi Ali M. S., Miskeen Elhadi, Hussein Kamal, Rezigalla Assad A., Adam Masoud I., Al-Faifi Jaber A., Mohammed Osama A. (2025). Evaluating the multiple-choice questions quality at the College of Medicine, University of Bisha, Saudi Arabia: A three-year experience. BMC Medical Education.

[B72-jintelligence-13-00102] El Masri Yasmine H., Ferrara Steve, Foltz Peter W., Baird Jo-Anne (2017). Predicting item difficulty of science national curriculum tests: The case of key stage 2 assessments. The Curriculum Journal.

[B74-jintelligence-13-00102] Embretson Susan (2023). Understanding examinees’ item responses through cognitive modeling of response accuracy and response times. Large-Scale Assessments in Education.

[B75-jintelligence-13-00102] Embretson Susan E. (1998). A cognitive design system approach to generating valid tests: Application to abstract reasoning. Psychological Methods.

[B76-jintelligence-13-00102] Embretson Susan E. (1999). Generating items during testing: Psychometric issues and models. Psychometrika.

[B77-jintelligence-13-00102] Embretson Susan E., Irvine Sidney, Kyllonen Patrick (2002). Generating abstract reasoning items with cognitive theory. Generating Items for Cognitive Tests: Theory and Practice.

[B78-jintelligence-13-00102] Embretson Susan E., Sternberg Robert J., Pretz Jean E. (2005). Measuring human intelligence with artificial intelligence. Cognition and Intelligence.

[B79-jintelligence-13-00102] Embretson Susan E. (2016). Understanding examinees’ responses to items: Implications for measurement. Educational Measurement: Issues and Practice.

[B80-jintelligence-13-00102] Embretson Susan E., Gorin Joanna S. (2001). Improving construct validity with cognitive psychology principles. Journal of Educational Measurement.

[B81-jintelligence-13-00102] Embretson Susan E., Kingston Neal M. (2018). Automatic item generation: A more efficient process for developing mathematics achievement items?. Journal of Educational Measurement.

[B82-jintelligence-13-00102] Embretson Susan E., Daniel Robert C. (2008). Understanding and quantifying cognitive complexity level in mathematical problem solving items. Psychology Science.

[B83-jintelligence-13-00102] Embretson Susan E., Yang Xiangdong, Rao Calyampudi R., Sinharay Sandip (2007). Automatic item generation and cognitive psychology. Handbook of Statistics: Vol 26 Psychometrics.

[B84-jintelligence-13-00102] Emekli Emre, Karahan Betül N. (2025). Comparison of automatic item generation methods in the assessment of clinical reasoning skills. Revista Española de Educación Médica.

[B85-jintelligence-13-00102] Enright Mary K., Morley Mary, Sheehan Kathleen M. (2002). Items by design: The impact of systematic feature variation on item statistical characteristics. Applied Measurement in Education.

[B86-jintelligence-13-00102] Estrada Eduardo, Ferrer Emilio, Abad Fransisco J., Román Fransisco J., Colom Roberto (2015). A general factor of intelligence fails to account for changes in tests’ scores after cognitive practice: A longitudinal multi-group latent variable study. Intelligence.

[B87-jintelligence-13-00102] Falcão Filipe, Pereira Daniela M., Gonçalves Nuno, De Champlain Andre, Costa Patrício, Pêgo José M. (2023). A suggestive approach for assessing item quality, usability and validity of Automatic Item Generation. Advances in Health Sciences Education.

[B88-jintelligence-13-00102] Falcão Filipe M. V., Pereira Daniela M., Pêgo José M., Costa Patrício (2024). Progress is impossible without change: Implementing automatic item generation in medical knowledge progress testing. Education and Information Technologies.

[B89-jintelligence-13-00102] Farrell Simon, Lewandowsky Stephan (2010). Computational models as aids to better reasoning in psychology. Current Directions in Psychological Science.

[B90-jintelligence-13-00102] Fehringer Benedict C. (2020). Spatial thinking from a different view: Disentangling top-down and bottom-up processes using eye tracking. Open Psychology.

[B91-jintelligence-13-00102] Fehringer Benedict C. (2023). Different perspectives on retest effects in the context of spatial thinking: Interplay of behavioral performance, cognitive processing, and cognitive workload. Journal of Intelligence.

[B92-jintelligence-13-00102] Fischer Gerhard H., Fischer G. H., Molenaar I. W. (1995). The Linear Logistic Test Model. Rasch Models. Foundations, Recent Developments, and Applications.

[B93-jintelligence-13-00102] Folk Valerie G., Smith Robert L., Mills Craig, Potenza Maria, Fremer John, Ward William (2002). Models for delivery of CBTs. Computer-Based Testing: Building the Foundation for Future Assessments.

[B94-jintelligence-13-00102] Foster David, Drasgow Fritz (2016). Testing technology and its effects on test security. Technology and Testing: Improving Educational and Psychological Measurement.

[B95-jintelligence-13-00102] Förster Natalie, Kuhn Jörg-Tobias (2023). Ice is hot and water is dry: Developing equivalent reading tests using rule-based item design. European Journal of Psychological Assessment.

[B96-jintelligence-13-00102] Freund Philipp A., Holling Heinz (2011). How to get real smart: Modeling retest and training effects in ability testing using computer-generated figural matrices items. Intelligence.

[B97-jintelligence-13-00102] Freund Philipp A., Hofer Stefan, Holling Heinz (2008). Explaining and controlling for the psychometric properties of computer-generated figural matrix items. Applied Psychological Measurement.

[B98-jintelligence-13-00102] Fried Eiko I. (2020). Lack of theory building and testing impedes progress in the factor and network literature. Psychological Inquiry.

[B99-jintelligence-13-00102] Fu Yanyan, Choe Edison M., Lim Hwanggyu, Choi Jaehwa (2022). An evaluation of automatic item generation: A case study of weak theory approach. Educational Measurement: Issues and Practice.

[B100-jintelligence-13-00102] Funk Paul F., Hoch Cosima C., Knoedler Samuel, Knoedler Leonard, Cotofana Sebastian, Sofo Giuseppe, Alfertshofer Michael (2024). ChatGPT’s response consistency: A study on repeated queries of medical examination questions. European Journal of Investigation in Health, Psychology and Education.

[B101-jintelligence-13-00102] Geerlings Hanneke, van der Linden Wim J., Glas Cees A. (2013). Optimal test design with rule-based item generation. Applied Psychological Measurement.

[B102-jintelligence-13-00102] Georgiadou Elissavet, Triantafillou Evangelos, Economides Anastasios A. (2007). A review of item exposure control strategies for computerized adaptive testing developed from 1983 to 2005. Journal of Technology, Learning, and Assessment.

[B103-jintelligence-13-00102] Gierl Mark J., Lai Hollis (2012). The role of item models in automatic item generation. International Journal of Testing.

[B104-jintelligence-13-00102] Gierl Mark J., Lai Hollis, Turner Simon (2012). Using automatic item generation to create multiple-choice items for assessments in medical education. Medical Education.

[B105-jintelligence-13-00102] Gierl Mark J., Shin Jinnie, Firoozi Tahereh, Lai Hollis (2022a). Using content coding and automatic item generation to improve test security. Frontiers in Education.

[B106-jintelligence-13-00102] Gierl Mark J., Swygert Kimberly, Matovinovic Donna, Kulesher Allison, Lai Hollis (2022b). Three sources of validation evidence needed to evaluate the quality of generated test items for medical licensure. Teaching and Learning in Medicine.

[B107-jintelligence-13-00102] Glas Cees A., van der Linden Wim J., Geerlings Hanneke, van der Linden Wim J. (2016). Item-family models. Handbook of Item Response Theory.

[B108-jintelligence-13-00102] Glas Cees A. W., van der Linden Wim J. (2003). Computerized adaptive testing with item cloning. Applied Psychological Measurment.

[B109-jintelligence-13-00102] Gorin Joanna S. (2005). Manipulating processing difficulty of reading comprehension questions: The feasibility of verbal item generation. Journal of Educational Measurement.

[B110-jintelligence-13-00102] Gorin Joanna S. (2006). Test design with cognition in mind. Educational Measurement: Issues and Practice.

[B111-jintelligence-13-00102] Gorin Joanna S., Embretson Susan E. (2006). Item diffficulty modeling of paragraph comprehension items. Applied Psychological Measurement.

[B112-jintelligence-13-00102] Graf Edith A., Peterson Stephen, Steffen Manfred, Lawless René (2005). Psychometric and Cognitive Analysis as a Basis for the Design and Revision of Quantitative Item Models (No. RR-05-25).

[B113-jintelligence-13-00102] Greeno James G., Moore Joyce L., Smith David R., Detterman Douglas K., Sternberg Robert J. (1993). Transfer of situated learning. Transfer on Trial: Intelligence, Cognition, and Instruction.

[B114-jintelligence-13-00102] Guest Olivia, Martin Andrea E. (2021). How computational modeling can force theory building in psychological science. Perspectives on Psychological Science.

[B115-jintelligence-13-00102] Gühne Daniela, Doebler Philipp, Condon David M., Luo Fang, Sun Luning (2020). Validity and reliability of automatically generated propositional reasoning items. European Journal of Psychological Assessment.

[B116-jintelligence-13-00102] Guo Jing, Tay Louis, Drasgow Fritz (2009). Conspiracy and test compromise: An evaluation of the resistance of test systems to small-scale cheating. International Journal of Testing.

[B117-jintelligence-13-00102] Gupta Piyush, Meena Pinky, Khan Amir M., Malhotra Rajeev K., Singh Tejinder (2020). Effect of faculty training on quality of multiple-choice questions. International Journal of Applied and Basic Medical Research.

[B118-jintelligence-13-00102] Hao Jiangang, von Davier Alina A., Yaneva Victoria, Lottridge Susan, von Davier Matthias, Harris Deborah J. (2024). Transforming assessment: The impacts and implications of large language models and generative AI. Educational Measurement: Issues and Practice.

[B119-jintelligence-13-00102] Hausknecht John P., Halpert Jane A., DiPaolo Nicole T., Moriarty Gerrard Meghan O. (2007). Retesting in selection: A meta-analysis of coaching and practice effects for tests of cognitive ability. Journal of Applied Psychology.

[B120-jintelligence-13-00102] Hayes Taylor R., Petrov Alexander A., Sederberg Per B. (2015). Do we really become smarter when our fluid intelligence test scores improve?. Intelligence.

[B121-jintelligence-13-00102] He Wei, Reckase Mark D. (2014). Item pool design for an operational variable-length computerized adaptive test. Educational and Psychological Measurement.

[B122-jintelligence-13-00102] Heil Martin, Jansen-Osmann Petra (2008). Sex differences in mental rotation with polygons of different complexity: Do men utilize holistic processes whereas women prefer piecemeal ones?. The Quarterly Journal of Experimental Psychology.

[B123-jintelligence-13-00102] Heil Martin, Rösler Frank, Link Michael, Bajric Jasmin (1998). What is improved if mental rotation task is repeated: The efficiency of memory access, or the speed of transformation routine?. Psychological Research.

[B124-jintelligence-13-00102] Hermes Michael, Albers Frank, Böhnke Jan R., Huelmann Gerrit, Maier Julia, Stelling Dirk (2019). Measurement and structural invariance of cognitive ability tests after computer-based training. Computers in Human Behavior.

[B125-jintelligence-13-00102] Hermes Michael, Maier Julia, Mittelstädt Justin, Albers Frank, Huelmann Gerrit, Stelling Dirk (2023). Computer-based training and repeated test performance: Increasing assessment fairness instead of retest effects. European Journal of Work and Organizational Psychology.

[B126-jintelligence-13-00102] Heston Thomas F., Khun Charya (2023). Prompt engineering in medical education. International Medical Education.

[B127-jintelligence-13-00102] Hickman Luis, Dunlop Patrick D., Wolf Jasper L. (2024). The performance of large language models on quantitative and verbal ability tests: Initial evidence and implications for unproctored high-stakes testing. International Journal of Selection and Assessment.

[B128-jintelligence-13-00102] Hines Scott (2017). The Development and Validation of an Automatic-Item Generation Measure of Cognitive Ability. Ph.D. dissertation.

[B129-jintelligence-13-00102] Holling Heinz, Bertling Jonas P., Zeuch Nina (2009). Automatic item generation of probability word problems. Studies in Educational Evaluation.

[B130-jintelligence-13-00102] Holmes Stephen D., Meadows Michelle, Stockford Ian, He Qingping (2018). Investigating the comparability of examination difficulty using comparative judgement and Rasch modelling. International Journal of Testing.

[B131-jintelligence-13-00102] Hornke Lutz F., Habon Michael W. (1986). Rule-based item bank construction and evaluation within the linear logistic framework. Applied Psychological Measurement.

[B132-jintelligence-13-00102] Impara James C., Plake Barbara S. (1998). Teachers’ ability to estimate item difficulty: A test of the assumptions in the Angoff standard setting method. Journal of Educational Measurement.

[B133-jintelligence-13-00102] Irvine Sidney H., Irvine Sidney H., Kyllonnen Patrick C. (2002). The foundations of item generation for mass testing. Item Generation for Test Development.

[B134-jintelligence-13-00102] Irvine Sidney H., Kyllonen Patrick C. (2002). Item Generation for Test Development.

[B135-jintelligence-13-00102] Ivie Jennifer L., Embretson Susan E. (2010). Cognitive process modeling of spatial ability: The assembling objects task. Intelligence.

[B136-jintelligence-13-00102] Jarosz Andrew F., Wiley Jennifer (2012). Why does working memory capacity predict RAPM performance? A possible role of distraction. Intelligence.

[B137-jintelligence-13-00102] Joncas Sébastien X., St-Onge Christina, Bourque Sylvie, Farand Paul (2018). Re-using questions in classroom-based assessment: An exploratory study at the undergraduate medical education level. Perspectives on Medical Education.

[B138-jintelligence-13-00102] Jozefowicz Ralph F., Koeppen Bruce M., Case Susan, Galbraith Robert, Swanson David, Glew Robert H. (2002). The quality of in-house medical school examinations. Academic Medicine.

[B139-jintelligence-13-00102] Kaller Christoph P., Rahm Benjamin, Köstering Lena, Unterrainer Josef M. (2011). Reviewing the impact of problem structure on planning: A software tool for analyzing tower tasks. Behavioural Brain Research.

[B140-jintelligence-13-00102] Kamruzzaman Mahammed, Nguyen Hieu, Hassan Nazmul, Kim Gene L. (2024). “A Woman is More Culturally Knowledgeable than A Man?”: The Effect of Personas on Cultural Norm Interpretation in LLMs. arXiv.

[B141-jintelligence-13-00102] Kapoor Radhika, Truong Sang T., Haber Nick, Ruiz-Primo Maria A., Domingue Benjamin W. (2025). Prediction of item difficulty for reading comprehension items by creation of annotated item repository. arXiv.

[B142-jintelligence-13-00102] Kara Basek Erdem, Dogan Nuri (2022). The effect of ratio of items indicating differential item functioning on computer adaptive and multi-stage tests. International Journal of Assessment Tools in Education.

[B143-jintelligence-13-00102] Karthikeyan Sowmiya, O’Connor Elizabeth, Hu Wendy (2019). Barriers and facilitators to writing quality items for medical school assessments–a scoping review. BMC Medical Education.

[B144-jintelligence-13-00102] Kıyak Yavuz S., Kononowicz Andrzej A. (2025). Using a hybrid of AI and template-based method in automatic item generation to create multiple-choice questions in medical education: Hybrid AIG. JMIR Formative Research.

[B145-jintelligence-13-00102] Kıyak Yavuz S., Kononowicz Andrzej A., Górski Stanislaw (2024). Multilingual template-based automatic item generation for medical education supported by generative artificial intelligence models ChatGPT and Claude. Bio-Algorithms and Med-Systems.

[B146-jintelligence-13-00102] Kıyak Yavuz S., Emekli Emre, Coşkun Özlem, Budakoğlu Işil İ (2025). Keeping humans in the loop efficiently by generating question templates instead of questions using AI: Validity evidence on Hybrid AIG. Medical Teacher.

[B147-jintelligence-13-00102] Klahr David, MacWhinney Brian, Damon William, Kuhn Deanna, Siegler Robert (1997). Information Processing. Cognition, Perception, and Language. Handbook of Child Psychology.

[B148-jintelligence-13-00102] Kosh Audra E., Simpson Mary A., Bickel Lisa, Kellogg Mark, Sanford-Moore Ellie (2019). A cost–benefit analysis of automatic item generation. Educational Measurement: Issues and Practice.

[B149-jintelligence-13-00102] Krautter Kai, Lehmann Jessica, Kleinort Eva, Koch Marco, Spinath Frank M., Becker Nicolas (2021). Test preparation in figural matrices tests: Focus on the difficult rules. Frontiers in Psychology.

[B150-jintelligence-13-00102] Kulik James A., Kulik Chen-Lin C., Bangert Robert L. (1984a). Effects of practice on aptitude and achievement test scores. American Educational Research Journal.

[B151-jintelligence-13-00102] Kulik James A., Bangert-Drowns Robert L., Kulik Chen-Lin C. (1984b). Effectiveness of coaching for aptitude tests. Psychological Bulletin.

[B152-jintelligence-13-00102] Kurdi Ghader, Leo Jared, Parsia Bijan, Sattler Uli, Al-Emari Salam (2020). A systematic review of automatic question generation for educational purposes. International Journal of Artificial Intelligence in Education.

[B153-jintelligence-13-00102] LaDuca Anthony, Staples William I., Templeton Bryce, Holzman Gerald B. (1986). Item modelling procedures for constructing content-equivalent multiple-choice questions. Medical Education.

[B154-jintelligence-13-00102] Lai Hollis, Gierl Mark J., Touchie Claire, Pugh Debra, Boulais André-Philippe, De Champlain André (2016). Using automatic item generation to improve the quality of MCQ distractors. Teaching and Learning in Medicine.

[B155-jintelligence-13-00102] Lane Suzanne, Raymond Mark, Haladyna Thomas, Lane Suzanne, Raymond Mark, Haladyna Thomas (2016). Test development process. Handbook of Test Development.

[B156-jintelligence-13-00102] Lang Jonas W. B., Hornke Lutz F., Amelang Manfred, Kersting Martin (2011). Computer-adaptives Testen. Enzyklopädie für Psychologie: Verfahren zur Leistungs-, Intelligenz- und Verhaltensdiagnostik.

[B157-jintelligence-13-00102] Lee Hye Y., Yune So J., Lee Sang Y., Im Sunju, Kam Bee S. (2024). The impact of repeated item development training on the prediction of medical faculty members’ item difficulty index. BMC Medical Education.

[B158-jintelligence-13-00102] Lee Jennifer C., Quadlin Natasha, Ambriz Denise (2023a). Shadow education, pandemic style: Social class, race, and supplemental education during COVID-19. Research in Social Stratification and Mobility.

[B159-jintelligence-13-00102] Lee Jooyoung, Le Thai, Chen Jinghui, Lee Dongwon (2022). Do language models plagiarize?. arXiv.

[B160-jintelligence-13-00102] Lee Philseok, Fyffe Shea, Son Mina, Jia Zihao, Yao Ziyu (2023b). A paradigm shift from “human writing” to “machine generation” in personality test development: An application of state-of-the-art natural language processing. Journal of Business and Psychology.

[B161-jintelligence-13-00102] Lee Unggi, Jung Haewon, Jeon Younghoon, Sohn Younghoon, Hwang Wonhee, Moon Jewoong, Kim Hyeoncheol (2023c). Few-shot is enough: Exploring ChatGPT prompt engineering method for automatic question generation in English education. Education and Information Technologies.

[B162-jintelligence-13-00102] Leslie Tara, Gierl Mark J. (2023). Using automatic item generation to create multiple-choice questions for pharmacy assessment. American Journal of Pharmaceutical Education.

[B163-jintelligence-13-00102] Levacher Julie, Koch Marco, Hissbach Johanna, Spinath Frank M., Becker Nicolas (2021). You can play the game without knowing the rules-but you’re better off knowing them: The influence of rule knowledge on figural matrices tests. European Journal of Psychological Assessment.

[B164-jintelligence-13-00102] Li Kunze, Zhang Yu (2024). Planning first, question second: An LLM-guided method for controllable question generation. Findings of the Association for Computational Linguistics ACL 2024.

[B165-jintelligence-13-00102] Lievens Filip, Reeve Charlie L., Heggestad Eric D. (2007). An examination of psychometric bias due to retesting on cognitive ability tests in selection settings. Journal of Applied Psychology.

[B166-jintelligence-13-00102] Lievens Filip, Buyse Tine, Sackett Paul R. (2005). Retest effects in operational selection settings: Development and test of a framework. Personnel Psychology.

[B167-jintelligence-13-00102] Lilly Jane, Montgomery Paul (2011). Systematic reviews of the effects of preparatory courses on university entrance examinations in high school-age students. International Journal of Social Welfare.

[B168-jintelligence-13-00102] Lim Sangdon, Choi Seung W. (2024). Item exposure and utilization control methods for optimal test assembly. Behaviormetrika.

[B169-jintelligence-13-00102] Lin Zhiqing, Chen Huilin (2024). Investigating the capability of ChatGPT for generating multiple-choice reading comprehension items. System.

[B170-jintelligence-13-00102] Liu Cheng, Han Kyung T., Li Jun (2019). Compromised item detection for computerized adaptive testing. Frontiers in Psychology.

[B171-jintelligence-13-00102] Liu Mingxin, Okuhara Tsuyoshi, Chang XinYi, Shirabe Ritsuko, Nishiie Yuriko, Okada Hiroko, Kiuchi Takahiro (2024). Performance of ChatGPT across different versions in medical licensing examinations worldwide: Systematic review and meta-analysis. Journal of Medical Internet Research.

[B172-jintelligence-13-00102] Liu Pengfei, Yuan Weizhe, Fu Jinlan, Jiang Zhengbao, Hayashi Hiroaki, Neubig Graham (2023a). Pre-train, prompt, and predict: A systematic survey of prompting methods in natural language processing. ACM Computing Surveys.

[B173-jintelligence-13-00102] Liu Yaohui, He Keren, Man Kaiwen, Zhan Peida (2025). Exploring critical eye-tracking metrics for identifying cognitive strategies in Raven’s Advanced Progressive Matrices: A data-driven perspective. Journal of Intelligence.

[B174-jintelligence-13-00102] Liu Yaohui, Zhan Peida, Fu Yanbin, Chen Qipeng, Man Kaiwen, Luo Yikun (2023b). Using a multi-strategy eye-tracking psychometric model to measure intelligence and identify cognitive strategy in Raven’s advanced progressive matrices. Intelligence.

[B175-jintelligence-13-00102] Loesche Patrick, Wiley Jennifer, Hasselhorn Marcus (2015). How knowing the rules affects solving the Raven Advanced Progressive Matrices Test. Intelligence.

[B176-jintelligence-13-00102] Lu Pan, Qiu Liang, Yu Wenhao, Welleck Sean, Chang Kai-Wei (2022). A survey of deep learning for mathematical reasoning. arXiv.

[B177-jintelligence-13-00102] Luca Massimiliano, Beneduce Ciro, Lepri Bruno, Staiano Jacopo (2025). The LLM wears Prada: Analysing gender bias and stereotypes through online shopping data. arXiv.

[B178-jintelligence-13-00102] Luecht Richard M. (2005). Some useful cost-benefit criteria for evaluating computer-based test delivery models and systems. Association of Test Publishers Journal.

[B179-jintelligence-13-00102] Matteucci Mariagiulia, Mignani Stefania, Veldkamp Bernard P. (2012). The use of predicted values for item parameters in item response theory models: An application in intelligence tests. Journal of Applied Statistics.

[B180-jintelligence-13-00102] Matton Nadine, Vautier Stéphane, Raufaste Éric (2011). Test-specificity of the advantage of retaking cognitive ability tests. International Journal of Selection and Assessment.

[B181-jintelligence-13-00102] McCoy R. Thomas, Smolensky Paul, Linzen Tal, Gao Jianfeng, Celikyilmaz Asli (2023). How much do language models copy from their training data? evaluating linguistic novelty in text generation using raven. Transactions of the Association for Computational Linguistics.

[B182-jintelligence-13-00102] Messick Samuel (1982). Issues of effectiveness and equity in the coaching controversy: Implications for educational testing and practice. Educational Psychologist.

[B183-jintelligence-13-00102] Messick Samuel, Jungeblut Ann (1981). Time and method in coaching for the SAT. Psychological Bulletin.

[B184-jintelligence-13-00102] Mislevy Robert J., Riconscente Michelle M., Downing Steven, Haladyna Thomas (2006). Evidence-centered assessment design: Layers, concepts, and terminology. Handbook of Test Development.

[B185-jintelligence-13-00102] Mislevy Robert J., Sheehan Kathleen M., Wingersky Marilyn (1993). How to equate tests with little or no data. Journal of Educational Measurement.

[B186-jintelligence-13-00102] Mislevy Robert J., Almond Russell G., Lukas Janice F. (2003). A Brief Introduction to Evidence-Centered Design (Research Report: RR-03-16).

[B187-jintelligence-13-00102] Morley Mary E., Bridgeman Brent, Lawless René R. (2004). Transfer Between Variants of Quantitative Items (GRE Board Rep. No. 00-06R).

[B188-jintelligence-13-00102] Nemec Eric C., Welch Beth (2016). The impact of a faculty development seminar on the quality of multiple-choice questions. Currents in Pharmacy Teaching and Learning.

[B190-jintelligence-13-00102] OpenAI (2023). GPT-4 technical report. arXiv.

[B189-jintelligence-13-00102] O’Reilly Tenaha, Feng Gary, Sabatini John, Wang Zuowei, Gorin Joanna (2018). How do people read the passages during a reading comprehension test? The effect of reading purpose on text processing behavior. Educational Assessment.

[B191-jintelligence-13-00102] Park Julie J., Becks Ann H. (2015). Who benefits from SAT prep? An examination of high school context and race/ethnicity. The Review of Higher Education.

[B192-jintelligence-13-00102] Piromsombat Chayut (2014). Differential Item Functioning in Computerized Adaptive Testing: Can CAT Self-Adjust Enough? (Publication No. 3620715). Doctoral dissertation.

[B193-jintelligence-13-00102] Powers Donald E. (2005). Effects of Pre-Examination Disclosure of Essay Prompts for the GRE Analytical Writing Assessment (Research Report: RR-05–01).

[B194-jintelligence-13-00102] Powers Donald E. (2012). Understanding the Impact of Special Preparation for Admissions Tests. Advancing Human Assessment: The Methodological, Psychological and Policy Contributions of ETS.

[B195-jintelligence-13-00102] Powers Donald E., Rock Donald A. (1999). Effects of coaching on SAT I: Reasoning scores. Journal of Educational Measurement.

[B196-jintelligence-13-00102] Powers Donald E., Alderman Donald L. (1983). Effects of test familiarization on SAT performance. Journal of Educational Measurement.

[B197-jintelligence-13-00102] Primi Ricardo (2002). Complexity of geometric inductive reasoning tasks: Contribution to the understanding of fluid intelligence. Intelligence.

[B198-jintelligence-13-00102] Primi Ricardo (2014). Developing a fluid intelligence scale through a combination of Rasch modeling and cognitive psychology. Psychological Assessment.

[B199-jintelligence-13-00102] Raffel Colin, Shazeer Noam, Roberts Adam, Lee Katherine, Narang Sharan, Matena Michael, Liu Peter J. (2020). Exploring the limits of transfer learning with a unified text-to-text transformer. The Journal of Machine Learning Research.

[B200-jintelligence-13-00102] Rajeb Mehdi, Krist Andrew T., Shi Qingzhou, Oyeniran Daniel O., Wind Stefanie A., Lakin Joni M. (2024). Mental rotation performance: Contribution of item features to difficulties and functional adaptation. Journal of Intelligence.

[B201-jintelligence-13-00102] Ranjan Rajesh, Gupta Shailja, Singh Saranyan N. (2024). Gender Biases in LLMs: Higher intelligence in LLM does not necessarily solve gender bias and stereotyping. arXiv.

[B202-jintelligence-13-00102] Reckase Mark D. (2010). Designing item pools to optimize the functioning of a computerized adaptive test. Psychological Test and Assessment Modeling.

[B203-jintelligence-13-00102] Reckase Mark D., Ju Unhee, Kim Sewon (2019). How adaptive is an adaptive test: Are all adaptive tests adaptive?. Journal of Computerized Adaptive Testing.

[B204-jintelligence-13-00102] Reeve Charlie L., Lam Holly (2005). The psychometric paradox of practice effects due to retesting: Measurement invariance and stable ability estimates in the face of observed score changes. Intelligence.

[B205-jintelligence-13-00102] Ren Xuezhu, Goldhammer Frank, Moosbrugger Helfried, Schweizer Karl (2012). How does attention relate to the ability-specific and position-specific components of reasoning measured by APM?. Learning and Individual Differences.

[B206-jintelligence-13-00102] Reynolds Laria, McDonell Kyle (2021). Prompt programming for large language models: Beyond the few-shot paradigm. Extended Abstracts of the 2021 CHI Conference on Human Factors in Computing Systems.

[B207-jintelligence-13-00102] Reza Mohi, Anastasopoulos Ioannis, Bhandari Shreya, Pardos Zachary A. (2024). PromptHive: Bringing subject matter experts back to the forefront with collaborative prompt engineering for educational content creation. arXiv.

[B208-jintelligence-13-00102] Riedel Maximilian, Kaefinger Katharina, Stuehrenberg Antonia, Ritter Viktoria, Amann Niklas, Graf Anna, Recker Florian, Klein Evelyn, Kiechle Marion, Riedel Fabian (2023). ChatGPT’s performance in German OB/GYN exams—Paving the way for AI-enhanced medical education and clinical practice. Frontiers in Medicine.

[B209-jintelligence-13-00102] Rogausch Anja, Hofer Rainer, Krebs René (2010). Rarely selected distractors in high stakes medical multiple-choice examinations and their recognition by item authors: A simulation and survey. BMC Medical Education.

[B210-jintelligence-13-00102] Roid Gale H., Haladyna Thomas M. (1982). Toward a Technology of Test-Item Writing.

[B211-jintelligence-13-00102] Runge Andrew, Attali Yigal, LaFlair Geoffrey T., Park Yena, Church Jaqueline (2024). A generative AI-driven interactive listening assessment task. Frontiers in Artificial Intelligence.

[B212-jintelligence-13-00102] Ryoo Ji H., Park Sunhee, Suh Hongwook, Choi Jaehwa, Kwon Jongkyum (2022). Development of a new measure of cognitive ability using automatic item generation and its psychometric properties. SAGE Open.

[B213-jintelligence-13-00102] Sahin Alper, Anil Duygu (2017). The effects of test length and sample size on item parameters in item response theory. Educational Science: Theory and Practice.

[B214-jintelligence-13-00102] Sahin Kursad Merve, Yalcin Seher (2024). Effect of differential item functioning on computer adaptive testing under different conditions. Applied Psychological Measurement.

[B215-jintelligence-13-00102] Sahoo Pranab, Singh Ayush K., Saha Sriparna, Jain Vinija, Mondal Samrat, Chadha Aman (2024). A systematic survey of prompt engineering in large language models: Techniques and applications. arXiv.

[B217-jintelligence-13-00102] Sayin Ayfer, Gierl Mark J. (2023). Automatic item generation for online measurement and evaluation: Turkish literature items. International Journal of Assessment.

[B218-jintelligence-13-00102] Sayin Ayfer, Gierl Marl (2024). Using OpenAI GPT to generate reading comprehension items. Educational Measurement: Issues and Practice.

[B219-jintelligence-13-00102] Sayın Ayfer, Bulut Okan (2024). The difference between estimated and perceived item difficulty: An empirical study. International Journal of Assessment Tools in Education.

[B220-jintelligence-13-00102] Sayın Ayfer, Gören Sebahat (2023). Comparing estimated and real item difficulty using multi-facet Rasch analysis. Journal of Measurement and Evaluation in Education and Psychology.

[B216-jintelligence-13-00102] Säuberli Andreas, Clematide Simon (2024). Automatic generation and evaluation of reading comprehension test items with large language models. arXiv.

[B221-jintelligence-13-00102] Scharfen Jana, Peters Judith M., Holling Heinz (2018). Retest effects in cognitive ability tests: A meta-analysis. Intelligence.

[B222-jintelligence-13-00102] Schneider Benedikt, Sparfeldt Jörn R. (2021a). How to get better: Taking notes mediates the effect of a video tutorial on number series. Journal of Intelligence.

[B223-jintelligence-13-00102] Schneider Benedikt, Sparfeldt Jörn R. (2021b). How to solve number series items: Can watching video tutorials increase test scores?. Intelligence.

[B224-jintelligence-13-00102] Schneider Benedikt, Becker Nicolas, Krieger Florian, Spinath Frank M., Sparfeldt Jörn R. (2020). Teaching the underlying rules of figural matrices in a short video increases test scores. Intelligence.

[B225-jintelligence-13-00102] Schroeders Ulrich, Achaa-Amankwaa Priscilla (2025). Developing NOVA: Next-generation open vocabulary assessment.

[B226-jintelligence-13-00102] Schroeders Ulrich, Gnambs Timo (2025). Sample-size planning in item-response theory: A tutorial. Advances in Methods and Practices in Psychological Science.

[B227-jintelligence-13-00102] Schulhoff Sander, Ilie Michael, Balepur Nishant, Kahadze Konstantine, Liu Amanda, Si Chenglei, Resnik Philip (2024). The prompt report: A systematic survey of prompting techniques. arXiv.

[B228-jintelligence-13-00102] Schulze Balhorn Lukas, Weber Jana M., Buijsman Stefan, Hildebrandt Julian R., Ziefle Martina, Schweidtmann Artur M. (2024). Empirical assessment of ChatGPT’s answering capabilities in natural science and engineering. Scientific Reports.

[B229-jintelligence-13-00102] Segall Daniel O. (2004). A sharing item response theory model for computerized adaptive testing. Journal of Educational and Behavioral Statistics.

[B230-jintelligence-13-00102] Selvi Hüseyin (2020). Should items and answer keys of small-scale exams be published?. Higher Education Studies.

[B231-jintelligence-13-00102] Shi Qingzhou, Wind Stefanie A., Lakin Joni M. (2023). Exploring the influence of item characteristics in a spatial reasoning task. Journal of Intelligence.

[B232-jintelligence-13-00102] Shin Jinnie, Gierl Mark J. (2022). Generating reading comprehension items using automated processes. International Journal of Testing.

[B233-jintelligence-13-00102] Shultz Benjamin, DiDomenico Robert J., Goliak Kristen, Mucksavage Jeffrey (2025). Exploratory assessment of GPT-4’s effectiveness in generating valid exam items in pharmacy education. American Journal of Pharmaceutical Education.

[B234-jintelligence-13-00102] Siegler Robert S. (1996). Emerging Minds: The Process of Change in Children’s Thinking.

[B235-jintelligence-13-00102] Sinharay Sandip (2017). Which statistic should be used to detect item pre-knowledge when the set of compromised items is known?. Applied Psychological Measurement.

[B236-jintelligence-13-00102] Sinharay Sandip, Johnson Matthew S. (2008). Use of item models in a large-scale admissions test: A case study. International Journal of Testing.

[B237-jintelligence-13-00102] Sinharay Sandip, Johnson Matthew S., Williamson David M. (2003). Calibrating item families and summarizing the results using family expected response functions. Journal of Educational and Behavioral Statistics.

[B238-jintelligence-13-00102] Smaldino Paul E. (2020). How to build a strong theoretical foundation. Psychological Inquiry.

[B239-jintelligence-13-00102] Sobieszek Adam, Price Tadeusz (2022). Playing games with AIs: The limits of GPT-3 and similar large language models. Minds and Machines.

[B240-jintelligence-13-00102] Someshwar Shonai (2024). Quality Control and the Impact of Variation and Prediction Errors on Item Family Design. Doctoral dissertation.

[B241-jintelligence-13-00102] Sommer Markus, Herle Margit, Häusler Joachim, Arendasy Martin, Ebner Georg, Fleck Günther (2009). Von TAVTMB zu ATAVT: Eine Anwendung der Automatisierten Itemgenerierung unter einschränkenden Rahmenbedingungen. Zweites Österreichisches Symposium für Psychologie im Militär.

[B242-jintelligence-13-00102] Sommer Markus, Arendasy Martin E., Punter Joachim F., Feldhammer-Kahr Martina, Rieder Anita (2025). Does test preparation mediate the effect of parents’ level of educational attainment on medical school admission test performance?. Intelligence.

[B243-jintelligence-13-00102] Song Yishen, Du Junlei, Zheng Qinhua (2025). Automatic item generation for educational assessments: A systematic literature review. Interactive Learning Environments.

[B244-jintelligence-13-00102] Stricker Lawrence J. (1984). Test disclosure and retest performance on the SAT. Applied Psychological Measurement.

[B245-jintelligence-13-00102] Su Mei-Chin, Lin Li-En, Lin Li-Hwa, Chen Yu-Chun (2024). Assessing question characteristic influences on ChatGPT’s performance and response-explanation consistency: Insights from Taiwan’s nursing licensing exam. International Journal of Nursing Studies.

[B246-jintelligence-13-00102] Sun Luning, Liu Yanan, Luo Fang (2019). Automatic generation of number series reasoning items of high difficulty. Frontiers in Psychology.

[B247-jintelligence-13-00102] Svetina Dubravka, Gorin Joanna, Tatsuoka Kikumi K. (2011). Defining and comparing the reading comprehension construct: A cognitive-psychometric modeling approach. International Journal of Testing.

[B248-jintelligence-13-00102] Sydorenko Tetyana (2011). Item writer judgments of item difficulty versus actual item difficulty: A case study. Language Assessment Quarterly.

[B249-jintelligence-13-00102] Tan Bin, Armoush Nour, Mazzullo Elisabetta, Bulut Okan, Gierl Mark J. (2024). A review of automatic item generation techniques leveraging large language models. EdArXiv.

[B250-jintelligence-13-00102] te Nijenhuis Jan, Vianen Annelies E. M., Flier Henk van der (2007). Score gains on g-loaded tests: No g. Intelligence.

[B251-jintelligence-13-00102] Thakur Vishesh (2023). Unveiling gender bias in terms of profession across LLMs: Analyzing and addressing sociological implications. arXiv.

[B252-jintelligence-13-00102] Tian Chen, Choi Jaehwa (2023). The impact of item model parameter variations on person parameter estimation in computerized adaptive testing with automatically generated items. Applied Psychological Measurement.

[B253-jintelligence-13-00102] Touvron Hugo, Lavril Thibaut, Izacard Gautier, Martinet Xavier, Lachaux Marie-Anne, Lacroix Thimothée, Lample Guillaume (2023). Llama: Open and efficient foundation language models. arXiv.

[B254-jintelligence-13-00102] van der Linden Wim J., Glas Cees A. (2010). Elements of Adaptive Testing.

[B255-jintelligence-13-00102] van der Maas Han L., Snoek Lukas, Stevenson Claire E. (2021). How much intelligence is there in artificial intelligence? A 2020 update. Intelligence.

[B256-jintelligence-13-00102] Vaswani Ashish, Shazeer Noam, Parmar Niki, Uszkoreit Jakob, Jones Llion, Gomez Aidan N., Kaiser Łukasz U., Polosukhin Illia, Guyon Isabelle, von Luxburg Ulrike, Bengio Samy, Wallach Hanna, Fergus Rob, Vishwanathan Vichy SVN, Garnett Roman (2017). Attention is all you need. Advances in Neural Information Processing Systems.

[B257-jintelligence-13-00102] Veerkamp Wim J., Glas Cees A. W. (2000). Detection of known items in adaptive testing with a statistical quality control method. Journal of Educational and Behavioral Statistics.

[B258-jintelligence-13-00102] Veldkamp Bernard P., van der Linden Wim J., van der Linden Wim J., Glas Cees A. W. (2010). Designing item pools for adaptive testing. Computerized Adaptive Testing: Theory and Practice.

[B259-jintelligence-13-00102] Verguts Tom, De Boeck Paul (2002). The induction of solution rules in Raven’s Progressive Matrices. European Journal of Cognitive Psychology.

[B260-jintelligence-13-00102] Vigneau François, Caissie André F., Bors Douglas A. (2006). Eye-movement analysis demonstrates strategic influences on intelligence. Intelligence.

[B261-jintelligence-13-00102] von Davier Matthias (2018). Automated item generation with recurrent neural networks. Psychometrika.

[B262-jintelligence-13-00102] von Davier Matthias (2019). Training Optimus prime, M.D.: Generating medical certification items by fine-tuning OpenAI’s gpt2 transformer model. arXiv.

[B263-jintelligence-13-00102] Wagner-Menghin Michaela, Preusche Ingrid, Schmidts Michael (2013). The effects of reusing written test items: A study using the Rasch model. ISRN Education.

[B264-jintelligence-13-00102] Wainer Howard, Irvine Sidney H., Kyllonen Patrick C. (2002). On the automatic generation of items: Some whens, whys and hows. Item Generation for Test Development.

[B265-jintelligence-13-00102] Waldock William J., Zhang Joe, Guni Ahmad, Nabeel Ahmad, Darzi Ara, Ashrafian Hutan (2024). The accuracy and capability of artificial intelligence solutions in health care examinations and certificates: Systematic review and meta-analysis. Journal of Medical Internet Research.

[B266-jintelligence-13-00102] Wancham Kittitas, Tangdhanakanond Kamonwan, Kanjanawasee Sirichai (2023). Development of the automatic item generation system for the diagnosis of misconceptions about force and laws of motion. Eurasia Journal of Mathematics, Science and Technology Education.

[B267-jintelligence-13-00102] Wang Yi, Zhou Qian, Ledo David (2024). StoryVerse: Towards co-authoring dynamic plot with LLM-based character simulation via narrative planning. Paper presented at 19th International Conference on the Foundations of Digital Games.

[B268-jintelligence-13-00102] Webb Emily M., Phuong Jonathan S., Naeger David M. (2015). Does educator training or experience affect the quality of multiple-choice questions?. Academic Radiology.

[B269-jintelligence-13-00102] Weppert Daniel, Amelung Dorothee, Escher Malvin, Troll Leander, Kadmon Martina, Listunova Lena, Montasser Jana (2023). The impact of preparatory activities on the largest clinical aptitude test for prospective medical students in Germany. Frontiers in Education.

[B270-jintelligence-13-00102] Witt Elizabeth A. (1993). Meta-analysis and the effects of coaching for aptitude tests. Paper presented at the Annual Meeting of the American Educational research Association.

[B271-jintelligence-13-00102] Wonde Shewatatek G., Tadesse Tefera, Moges Belay, Schauber Stefan K. (2024). Experts’ prediction of item difficulty of multiple-choice questions in the Ethiopian Undergraduate Medicine Licensure Examination. BMC Medical Education.

[B272-jintelligence-13-00102] Wood Timothy J. (2009). The effect of reused questions on repeat examinees. Advances in Health Sciences Education.

[B273-jintelligence-13-00102] Wood Timothy J., St-Onge Christina, Boulais André-Philippe, Blackmore David E., Maguire Thomas O. (2010). Identifying the unauthorized use of examination material. Evaluation and the Health Professions.

[B274-jintelligence-13-00102] Yang Eunbae B., Lee Myung A., Park Yoon S. (2018). Effects of test item disclosure on medical licensing examination. Advances in Health Sciences Education.

[B275-jintelligence-13-00102] Yang Jingfeng, Jin Hongye, Tang Ruixiang, Han Xiaotian, Feng Qizhang, Jiang Haoming, Hu Xia (2024). Harnessing the power of LLMs in practice: A survey on ChatGPT and beyond. ACM Transactions on Knowledge Discovery from Data.

[B276-jintelligence-13-00102] Yang Yuan, Kunda Mathilee (2023). Computational models of solving Raven’s Progressive Matrices: A comprehensive introduction. arXiv.

[B277-jintelligence-13-00102] Yang Yuan, Sanyal Deepayan, Michelson Joel, Ainooson James, Kunda Mathilee (2022). Automatic item generation of figural analogy problems: A review and outlook. arXiv.

[B278-jintelligence-13-00102] Yi Qing, Zhang Jinming, Chang Hua-Hua (2008). Severity of organized item theft in computerized adaptive testing: A simulation study. Applied Psychological Measurement.

[B279-jintelligence-13-00102] Yu Jiayuan (1994). Homogenity of problem solving strategies and the fitting of linear logistic model. Acta Psychologica Sinica.

[B280-jintelligence-13-00102] Zenisky April, Hambleton Ronald K., Luecht Richard M., van der Linden Wim J., Glas Cees A. (2010). Multistage testing: Issues, designs, and research. Elements of Adaptive Testing.

[B281-jintelligence-13-00102] Zha Daochen, Bhat Zaid P., Lai Kwei-Herng, Yang Fan, Jiang Zhimeng, Zhong Saochen, Hu Xiaben (2025). Data-centric artificial intelligence: A survey. ACM Computing Surveys.

[B282-jintelligence-13-00102] Zhang Jinming, Chang Hua-Hua (2005). The Effectiveness of Enhancing Test Security by Using Multiple Item Pools (ETS RR-05-19).

[B283-jintelligence-13-00102] Zhang Jinming, Chang Hua-Hua, Yi Qing (2012). Comparing single-pool and multiple-pool designs regarding test security in computerized testing. Behavior Research Methods.

[B284-jintelligence-13-00102] Zickar Michael J. (2020). Measurement development and evaluation. Annual Review of Organizational Psychology and Organizational Behavior.

[B285-jintelligence-13-00102] Zimmer Felix, Henninger Mirka, Debelak Rudolf (2024). Sample size planning for complex study designs: A tutorial for the mlpwr package. Behavior Research Methods.

[B286-jintelligence-13-00102] Zimmermann Stefan, Klusmann Dietrich, Hampe Wolfgang (2016). Are exam questions known in advance? Using local dependence to detect cheating. PLoS ONE.

[B287-jintelligence-13-00102] Zorowitz Samuel, Chierchia Gabriele, Blakemore Sarah-Jayne, Daw Nathaniel D. (2024). An item response theory analysis of the matrix reasoning item bank (MaRs-IB). Behavior Research Methods.

[B288-jintelligence-13-00102] Zu Jiyun, Choi Ikkyu, Hao Jiangang (2023). Automated distractor generation for fill-in-the-blank items using a prompt-based learning approach. Psychological Testing and Assessment Modeling.

[B289-jintelligence-13-00102] Zwick Rebecca (2002). Is the SAT a ‘wealth test’?. Phi Delta Kappan.

[B290-jintelligence-13-00102] Zwick Rebecca, Thayer Dorothy T., Wingersky Marilyn (1995). Effect of Rasch calibration on ability and DIF estimation in computer-adaptive tests. Journal of Educational Measurement.

